# Engineered Molecular Therapeutics Targeting Fibrin and the Coagulation System: a Biophysical Perspective

**DOI:** 10.1007/s12551-022-00950-w

**Published:** 2022-04-06

**Authors:** Fanny Risser, Ivan Urosev, Joanan López-Morales, Yang Sun, Michael A. Nash

**Affiliations:** 1 Institute of Physical Chemistry, Department of Chemistry, University of Basel, 4058 Basel, Switzerland; 2 Department of Biosystems Sciences and Engineering, ETH Zurich, 4058 Basel, Switzerland

**Keywords:** Fibrin, Therapeutics, Hemostasis, Antibodies, Polymers, Hydrogels

## Abstract

The coagulation cascade represents a sophisticated and highly choreographed series of molecular events taking place in the blood with important clinical implications. One key player in coagulation is fibrinogen, a highly abundant soluble blood protein that is processed by thrombin proteases at wound sites, triggering self-assembly of an insoluble protein hydrogel known as a fibrin clot. By forming the key protein component of blood clots, fibrin acts as a structural biomaterial with biophysical properties well suited to its role inhibiting fluid flow and maintaining hemostasis. Based on its clinical importance, fibrin is being investigated as a potentially valuable molecular target in the development of coagulation therapies. In this topical review, we summarize our current understanding of the coagulation cascade from a molecular, structural and biophysical perspective. We highlight single-molecule studies on proteins involved in blood coagulation and report on the current state of the art in directed evolution and molecular engineering of fibrin-targeted proteins and polymers for modulating coagulation. This biophysical overview will help acclimatize newcomers to the field and catalyze interdisciplinary work in biomolecular engineering toward the development of new therapies targeting fibrin and the coagulation system.

## Introduction

The multicomponent coagulation system is regulated by an interplay of prothrombotic and antithrombotic signals in the blood. These signals must maintain a delicate balance in order to achieve appropriate hemostasis, being capable of rapidly stopping excessive bleeding or rebleeding events following trauma, while simultaneously avoiding unwanted, disseminated or prolonged thrombus formation. In light of recent clinical and molecular physiological studies on proteins involved in the coagulation cascade, the classical model of the intrinsic and extrinsic coagulation pathways is being evaluated more closely. Dysregulation of coagulation in COVID-19 patients (Helms et al. 2020; Connors and Levy 2020; Klok et al. 2020) as well as vaccine recipients (Schultz et al. 2021) furthermore demonstrates the importance of coagulation physiology in the treatment and prevention of transmissible diseases. Given the importance of coagulation and its role in a broad range of pathological conditions, the development of engineered biological therapeutics targeting fibrin and the coagulation system is of high interest in molecular biosciences.

This review provides a current perspective on engineered coagulation therapies with a particular emphasis on molecular, structural and biophysical properties of fibrin. We begin by summarizing our current understanding of the cellular and molecular events taking place during primary and secondary hemostasis. We highlight single-molecule biophysical studies on proteins involved in coagulation, with an emphasis on structure and conformation-based mechanisms. We summarize what is known about fibrin clots from a soft mechanics perspective and describe how molecular features of fibrin give rise to emergent mechanical properties at the network level that are well adapted to the physiological role of fibrin in stopping fluid flow. After this description of native coagulation, we provide an overview of currently available procoagulant hemostatic therapies, sealants and adhesives for topical and systemic clinical use in humans. Finally, we review reports on the development of fibrin-targeted antibodies, peptides and polymers. With this review, we provide a broad overview at the intersection between molecular engineering and biophysical analysis methods applied to fibrin and other proteins involved in coagulation.

## Molecular physiology of coagulation and hemostasis

### Primary hemostasis

Primary hemostasis refers to the initial steps of the coagulation cascade encompassing platelet adhesion, activation and formation of a platelet plug at a wound site. Upon injury to the vessel wall, blood platelets (i.e., thrombocytes) adhere to exposed sub-endothelial matrix proteins, including von Willebrand factor (VWF), collagen and fibronectin (Lenting et al. 2012). Following platelet adhesion, activation and aggregation, a platelet plug forms at the injury site (Rana et al. 2019). The arrest of platelets is known to be dependent on shear stress. In veins and larger arteries where the shear rate is low or intermediate (< 1000 s^−1^), α_IIb_β3 integrins that engage fibrinogen adsorbed onto the surface of thrombi are involved in platelet aggregation. In the arterial microcirculation or regions of arterial stenosis, shear rates occur in a range of 1,000–10,000 s^−1^, and platelet-platelet interactions become progressively more VWF-dependent with both GpIbα (a mechanosensing platelet surface receptor) and integrin α_IIb_β3 playing important roles. Under pathological shear (> 10,000 s^−1^) at sites of acute vessel narrowing or sites of atherothrombosis, platelet aggregation is exclusively mediated by VWF-GpIbα adhesive bonds (Jackson et al. 2009). Here, we will focus on the biomechanical properties of VWF that facilitate its interaction with platelets under high shear stress.

### Von Willebrand factor

**Fig. 1:**
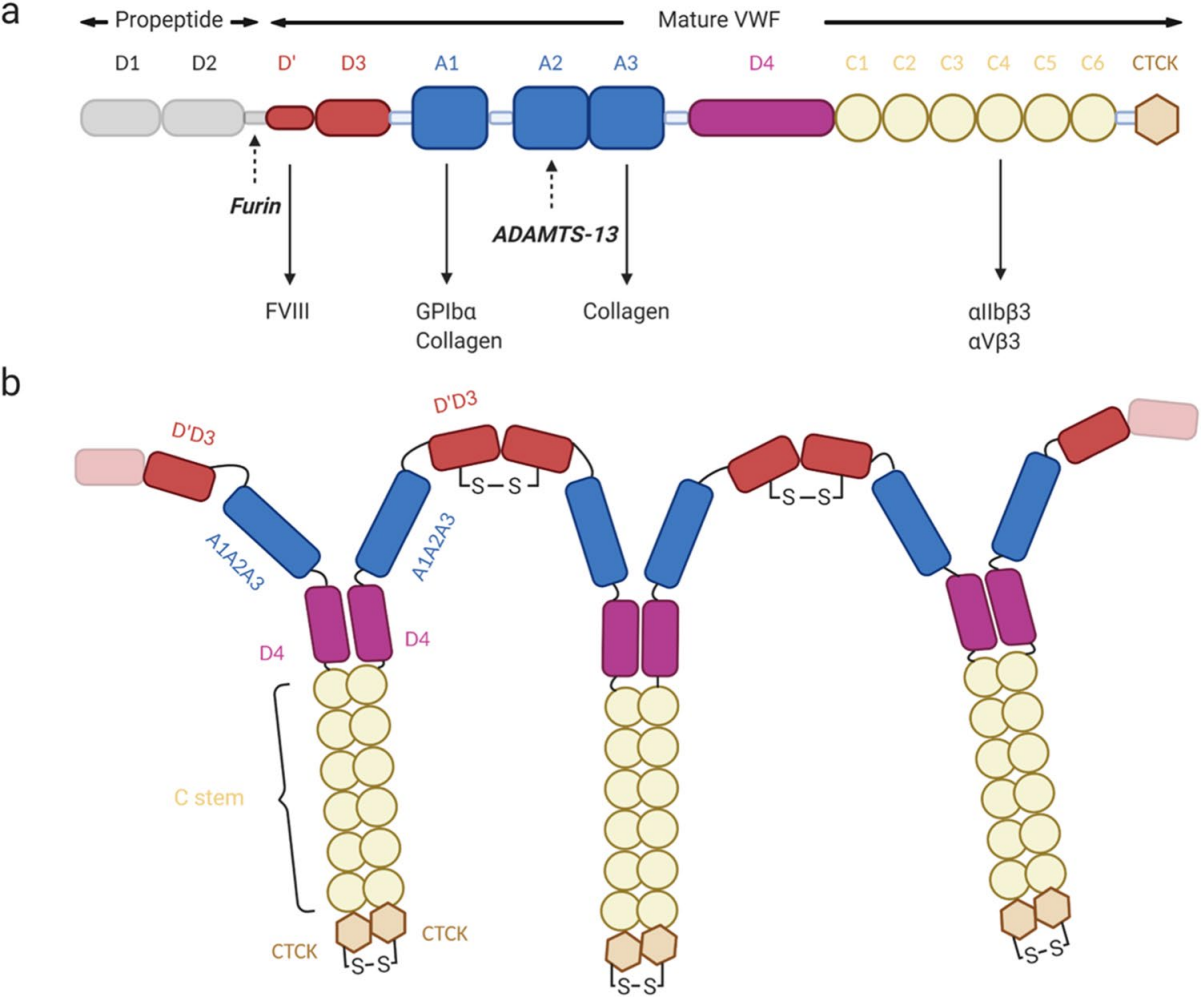
Schematic of von Willebrand Factor. (a) Domain annotation of a mature VWF monomer. (b) Schematic architecture of a VWF multimer linked through head-to-head and tail-to-tail disulfide bonds

Von Willebrand factor (VWF) is a multidomain protein made up of domains arranged in the order D1-D2-D’-D3-A1-A2-A3-D4-B1-B2-B3-C1-C6-CK (Fig. 1a), each with a specific structure and function (Zhou et al. 2012). Pro-VWF monomers associate in the endoplasmic reticulum, forming “tail-to-tail” dimers through C-terminal disulfide bonds on the CK domains, forming a quaternary structure referred to as a dimeric bouquet (Marti et al. 1987; Katsumi et al. 2000). Dimers then multimerize by forming “head-to-head” disulfide bonds between the N-terminal D3 domains in the Golgi (Fig. 1b) (Marti et al. 1987; Dong et al. 1994). VWF next undergoes post-translational modification through glycosylation and sulfation in the endoplasmic reticulum, Golgi and post-Golgi organelles. Additional modification occurs in the Golgi consisting of D1-D2 propeptide cleavage by furin (Sadler 1998; Springer 2014; Lancellotti et al. 2019).

A large portion (~ 95%) of VWF molecules are directly secreted into the bloodstream as low molecular weight multimers (Sporn et al. 1986). The remainder are stored in Weibel–Palade bodies of endothelial cells under the form of ultra large VWF (ULVWF), reaching up to 20,000 kDa, for stimulated secretion. Typically, tubule structures are adopted as shown by electron microscopy (Valentijn et al. 2011; Springer 2014). Other proteins involved in hemostasis and inflammation are also stored in the WPB, namely factor VIII (FVIII) that directly interacts with VWF’s D’ domain (Shiltagh et al. 2014). Release of the ULVWF concatemers and other proteins from WPB into the bloodstream happens by activation of secretagogues such as thrombin, histamines or Ca^2+^ (Valentijn et al. 2011). VWF secretion involves disassembly of the large helical structure adopted within the WPB and dimeric bouquet unzipping (Valentijn et al. 2011; Springer 2014). ULVWF concatemers secreted from endothelial cells are anchored to the cell surface and form extremely long threads that are rapidly cut in the presence of plasma from healthy donors (Dong et al. 2002), facilitating VWF release away from the membrane. Hence, circulating VWF adopts a variety of multimerization degrees, ranging from a 500 kDa population (corresponding to single dimers) to 10,000 kDa population (corresponding to 20 dimers) (Stockschlaeder et al. 2014).

**Fig. 2:**
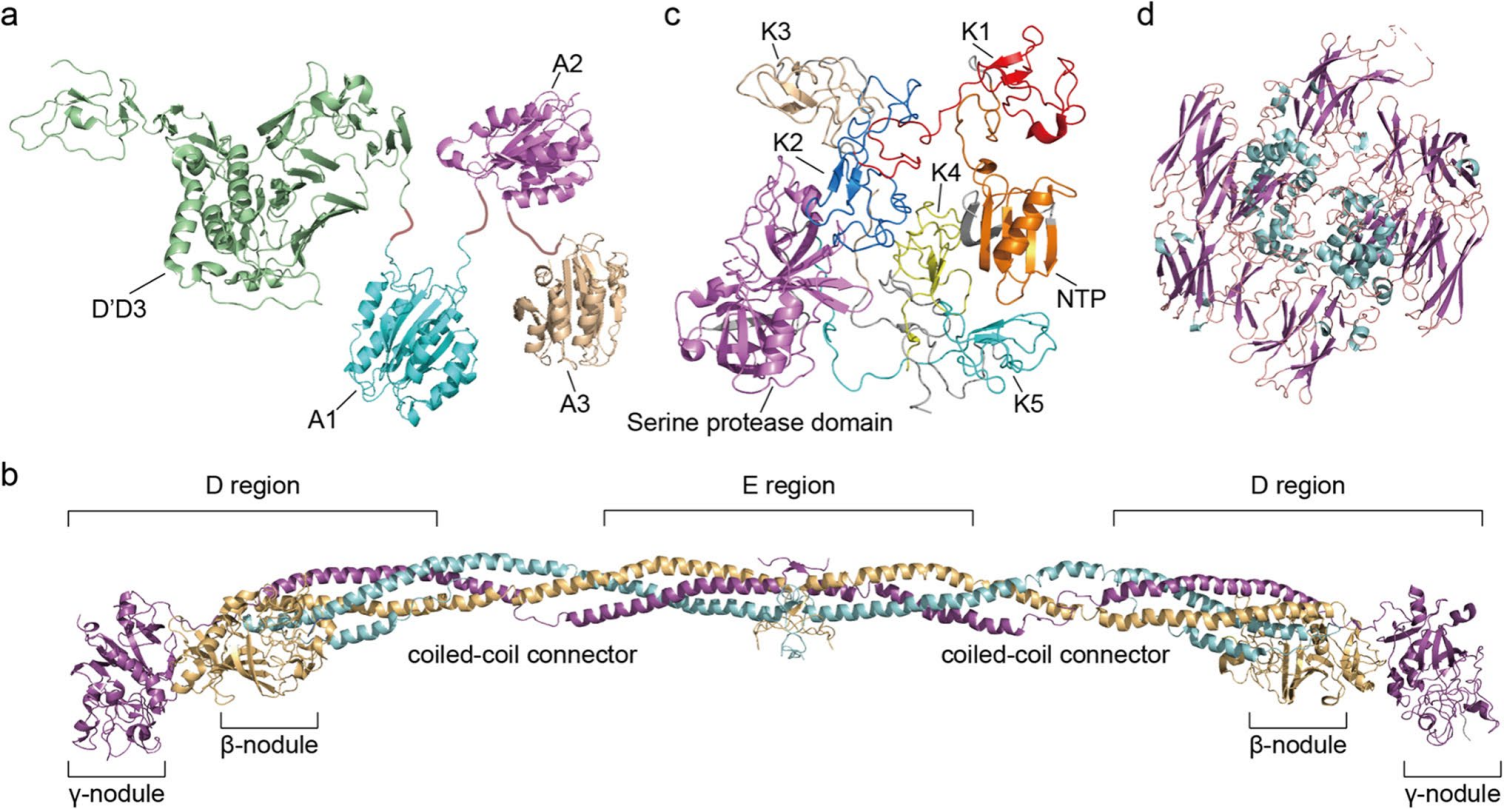
Diagram of crystal structures of proteins involved in coagulation. (a) Crystal structure of monomeric von Willebrand Factor D’D3 domain (PDB: 6N29, green), A1 domain (PDB: 1AUQ, cyan), A2 domain (PDB: 3GXB, violet) and A3 domain (PDB: 1AO3, wheat); (b) Crystal structure of human fibrinogen (PDB: 3GHG, α chain in cyan, β chain in wheat and γ chain in violet). (c) Crystal structure of full-length type II human plasminogen (PDB: 4DUR) (NTP in orange, K1 in red, K2 in blue, K3 in wheat, K4 in yellow, K5 in cyan, and serine protease domain in violet); (d) Crystal structure of recombinant human factor XIII a2 dimer (PDB: 1FIE) after cleavage by thrombin (α helix in cyan and β sheet in violet)

The primary function of VWF in the plasma is performed by the three adjacent A domains (Fig. 2a). A1 is responsible for binding to the platelet receptor GPIbα and collagen, A3 acts as the immobilization site for VWF on the collagen matrix in an ancillary manner, and A2 is responsible for size regulation of the concatemers. It is the main sensing domain that responds to shear stress (Springer 2014).

The other domains also play biochemical roles and do not only fill structural purposes. For instance, the C4 domain contains an RGD sequence that interacts with platelet receptor integrins α_IIb_β3 and αVβ3 (Fig. 1a). VWF functions therefore as a link between subendothelial collagen and platelets. As for D’, as mentioned earlier, it binds the procoagulant FVIII at an early stage, in the WPB. When VWF is secreted in the bloodstream, it acts as a carrier molecule and protects FVIII, increasing its plasma half-life and localizing it at the injury sites (Sadler 1998).

VWF is critically important both physiologically and pathologically, and dysfunction of VWF is responsible for several hemorrhagic disorders, including von Willebrand disease (VWD), a common hereditary disorder resulting from functional deficiencies in VWF. VWD presents in a range from mild to severe hemorrhagic episodes that can in the worst cases be fatal (Rana et al. 2019) with an overall incidence of approximately 1% in the general population.

### Role of mechanical forces in VWF activation

**Fig. 3:**
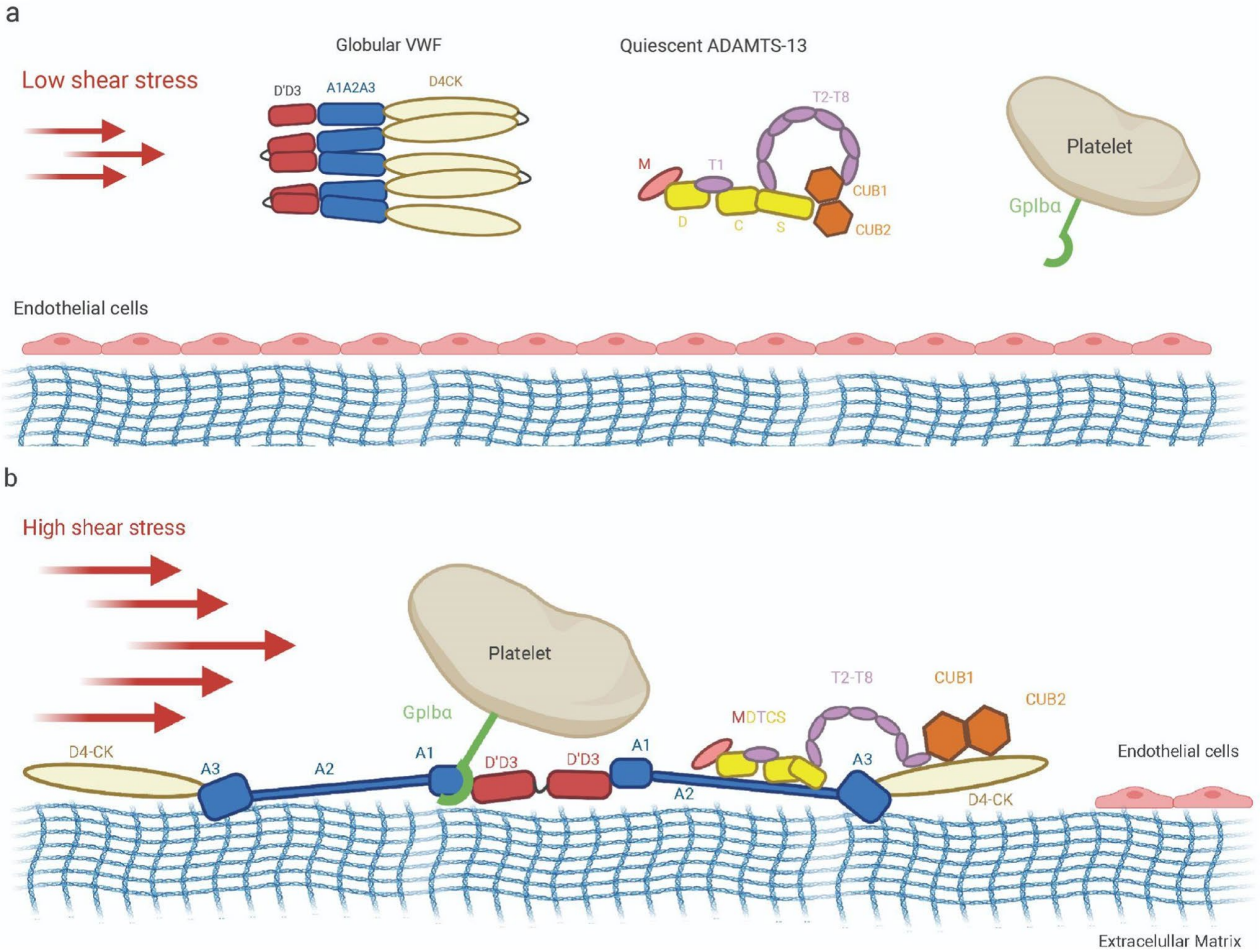
Schematic of shear-induced activation of multimeric VWF and subsequent interactions with exposed collagen, ADAMTS-13 and platelet GpIbα receptor. (a) Under low shear stress VWF adopts a compact conformation with the three A domains buried and inaccessible for binding. (b) In case of injury, shear stress increases due to vascular contraction, leading to mechanical unfolding of A2 and exposure of A1 and A3, revealing cryptic binding sites on VWF that interact with collagen, GPIbα and ADAMTS-13. ADAMTS-13 is conformationally activated upon interaction with VWF and cuts the unfolded A2 domain

VWF is directly regulated by hydrodynamic forces, and its activation involves force-induced conformational changes at the level of single domains and quaternary protein structure and conformation. Down-regulation of VWF-mediated responses is furthermore based on mechano-enzymatic cleavage at a cryptic binding site that becomes accessible upon A2 domain unfolding (Löf et al. 2018; Lancellotti et al. 2019). Schneider and colleagues showed that under low shear forces, VWF has a globular compact native conformation (Schneider et al. 2007) where the binding sites in the A1 and A3 domains are buried, sterically shielded by other domains and inaccessible for platelet binding (Fig. 3a). When subjected to hydrodynamic forces, VWF multimers undergo an abrupt transition to an extended conformation, which switches on binding activity to collagen (Fig. 3b). In the vasculature, VWF undergoes end-over-end tumbling as well as periodic elongation and compaction (Springer 2014; Rana et al. 2019). These stretching transitions can occur at sites of vascular injury following vasoconstriction or in stenosed vessels due to a reduction in vessel diameter and an increase in shear stress (Fig. 3b). These force-based signaling mechanisms rely on alterations to the normal blood flow profile in the vessel which increase elongational hydrodynamic forces acting on the VWF multimer (Rana et al. 2019; Kania et al. 2021).

Hydrodynamic forces increase nonlinearly with a molecule’s end-to-end contour length. Since mechanical unfolding transitions release contour length (i.e., biopolymer length increase upon unfolding), this can initiate a cascade of increasing force that triggers additional unfolding transitions in a positive feedback manner. Therefore, the larger the VWF multimers are, the more hemostatically active they are. Large VWF multimers (made of ~ 200 monomers) unfold at lower forces and bind collagen to recruit platelet more easily than smaller ones made of ~ 40 monomers (Zheng 2013; Stockschlaeder et al. 2014).

### A2 domain and ADAMTS-13

The A2 domain is the primary mechanosensitive domain of VWF. Specific structural properties such as lack of long-range disulfide linkages and Ca^2+^ binding make A2 distinct from A1 and A3, and hydrodynamic shear acts mostly on the A2 domain (Jakobi et al. 2011; Lancellotti et al. 2019). Single-molecule optical tweezers and atomic force microscope experiments demonstrated that A2 can be unfolded by elongational forces (Zhang et al. 2009; Müller et al. 2016b; Löf et al. 2019). A2 unfolding produces a length increment of roughly 45 nm (at 20 pN), which is approximately the same length of a VWF monomer. This unique sensitivity of A2 to shear stress is also an important aspect of ULVWF size regulation and therefore, hemostatic activity. A2 can be recognized and cut by the protease ADAMTS-13 (a disintegrin and metalloproteinase with thrombospondin motifs). The target site is located between Tyr1605 and Met1606 and is buried in the central β-sheet of the folded domain. Exposure of this cleaving site is achieved upon A2 domain unfolding. Since A2 can spontaneously refold when force is relaxed, the action of ADAMTS-13 is regulated by mechanical force and its influence on the folding state of A2 (Zhang et al. 2009). The requisite tensile force to unfold A2 corresponds to that experienced in the middle of a 200-mer VWF multimer in arterioles and capillaries or when VWF is bound to platelets, or collagen (Crawley et al. 2011). VWF has been shown to be more susceptible to cleavage by ADAMTS-13 when bound to platelets (Shim et al. 2008). Several studies carried out in recent years using truncation mutants and other approaches have shed light on the interaction and activation mechanisms between ADAMTS-13 and VWF that relies on structural transitions of A2 and of ADAMTS-13 from a quiescent, closed form to an active open form (Fig. 3b) (Muia et al. 2014; South et al. 2016, 2017; South and Lane 2018).

### VWF A1 and platelet GpIbα interaction

**Fig. 4:**
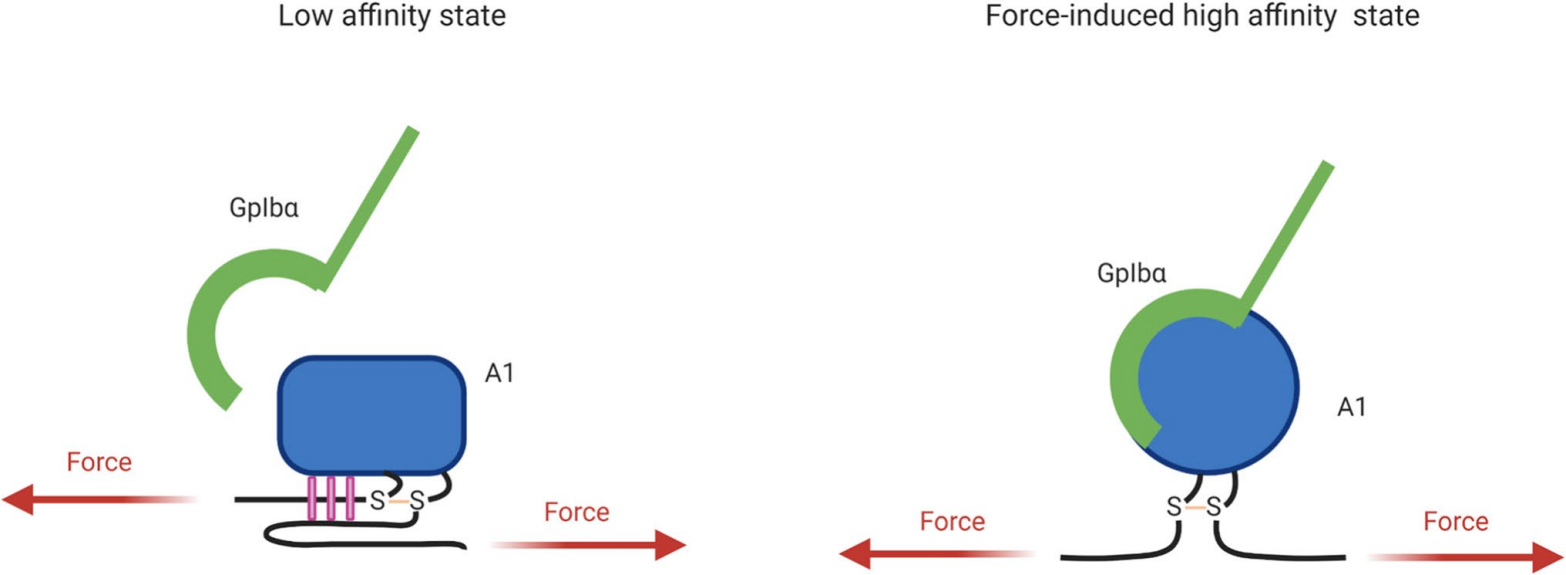
Model of force-induced activation of the A1-GpIbα interaction to a high affinity state. Mechanical stress applied between the N- and C-termini of A1 as well as from the platelet-bound GpIbα disrupts hydrogen bonds on residues external to the A1 disulfide bond, deforming the protein structure and transitioning the A1-GpIbα interaction from a low-affinity to a high-affinity conformation

**Fig. 5:**
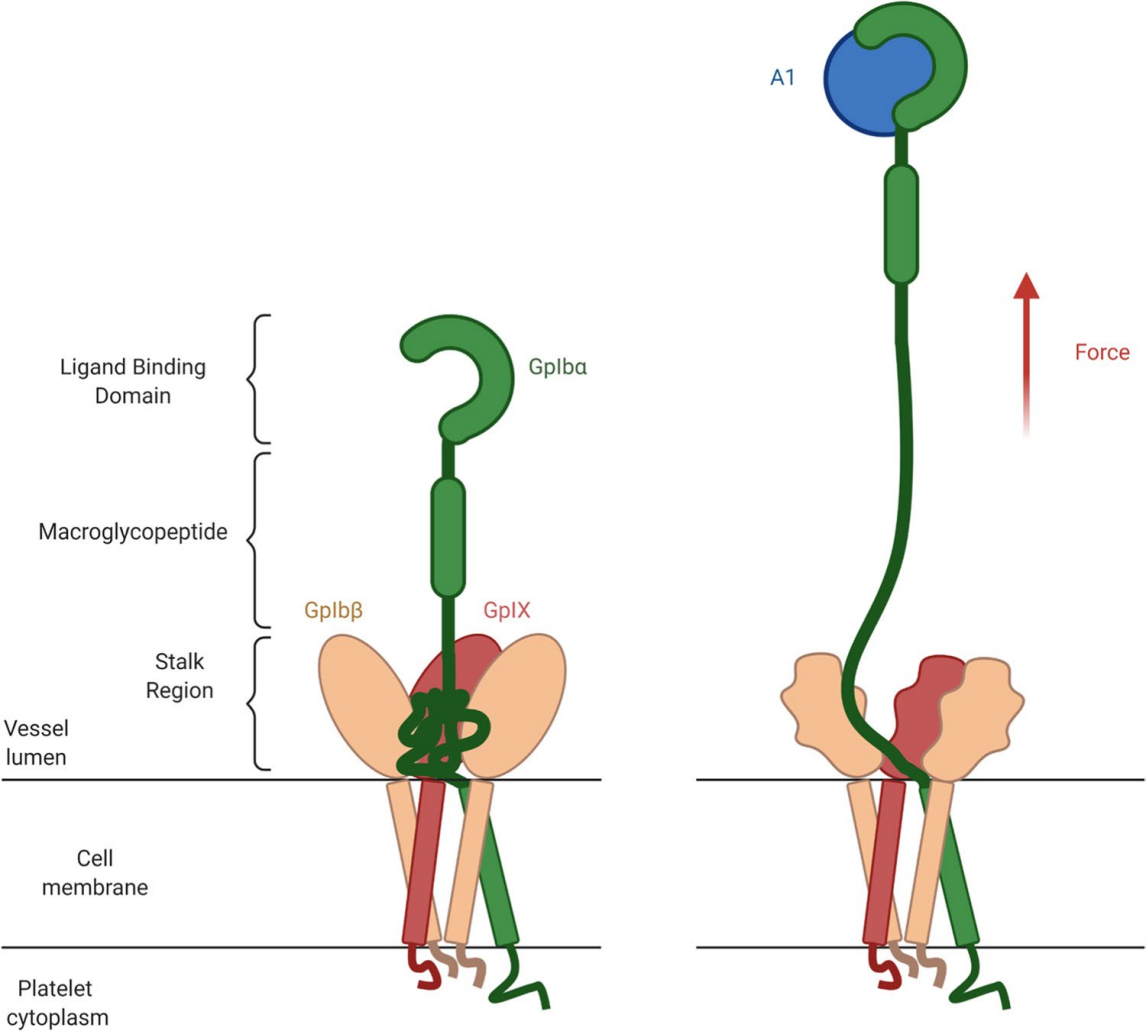
Mechanosensing mechanism of GpIb-IX. In the absence of shear forces, the stalk region is believed to be folded. Through VWF A1-mediated binding and exposure to mechanical tension, this region unfolds resulting in conformational changes to GpIb-IX that are propagated across the membrane to initiate signaling

In the last decade, researchers have studied the mechanical regulation of the interaction between VWF A1 and platelet receptor GpIbα by shear stress (Löf et al. 2018). At low shear stress, VWF concatemers adopt a loosely collapsed, globular conformation that prevents the A1-GpIbα interaction. Both short-range interactions between A1 and its flanking domains as well as long range interactions between various regions of VWF multimers are involved (Deng et al. 2017, 2018; Löf et al. 2018). Under elevated shear, VWF extends and mechanical stretching and unfolding of A1 exposes cryptic sites, enabling platelet binding (Fig. 3b) (Savage et al. 1996). The A1-GpIbα interaction was reported as having flex bond behavior, resulting from force-induced structural changes within A1 and/or GpIbα. A recent study on full length VWF described this force-dependent two-step activation mechanism (Fu et al. 2017). At low shear stress, VWF concatemers extend, leading to exposure of platelet binding sites contained within A1 domains. At this stage, A1 domains adopt a flexed, low affinity state toward GpIbα. Only at tension values > 20 pN, does A1 conformationally transition to a high affinity state, increasing the GpIbα–A1 bond lifetime. This unusual feature is believed to be responsible for platelet behaviors such as rolling, slowing, and stopping on the vessel wall (Figs. 4 and 5).

### Secondary hemostasis

**Fig. 6:**
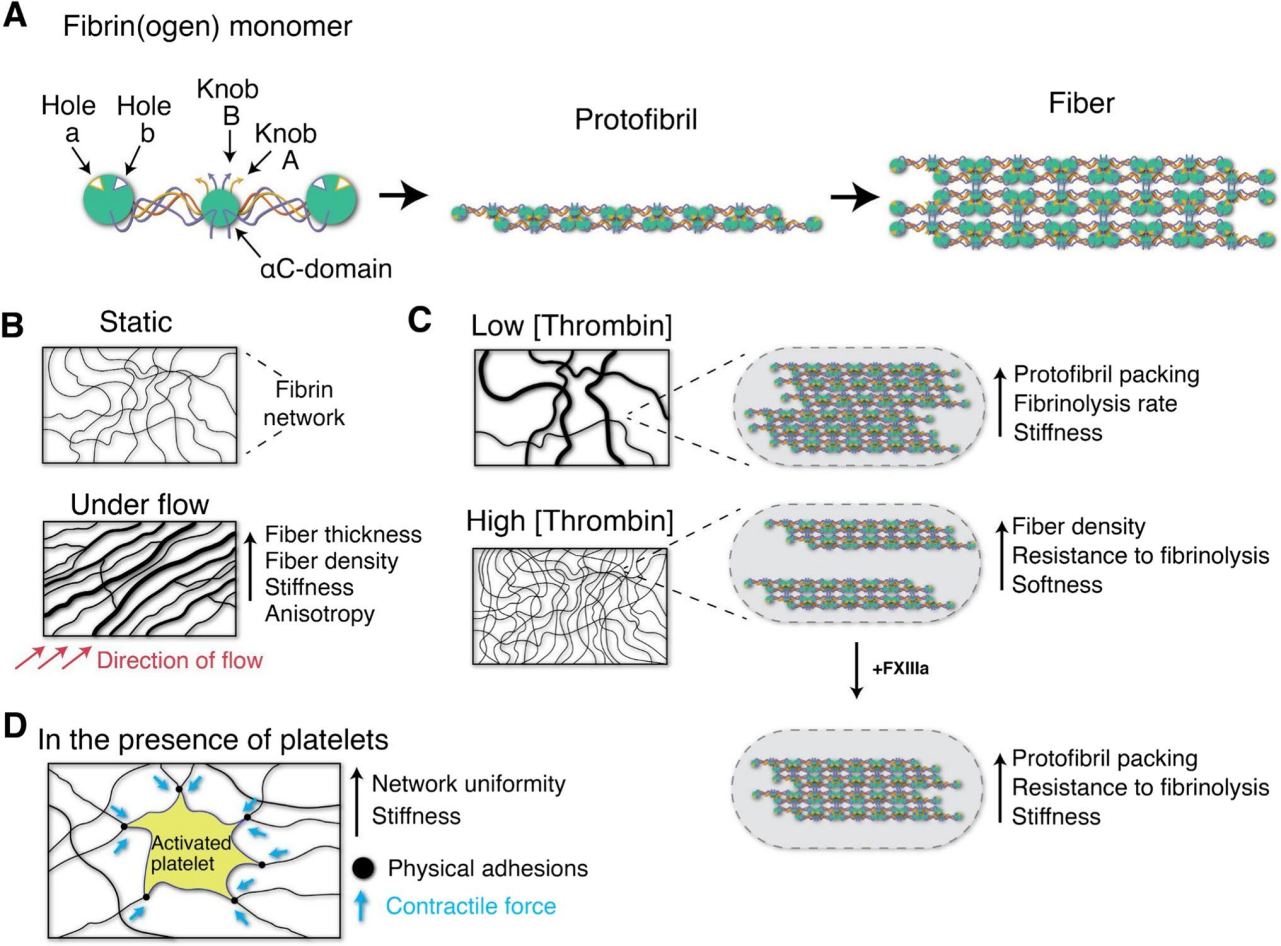
Overview of fibrin networks and modulation of fibrin properties. (a) Fibrinogen monomers are cleaved by thrombin protease revealing knobs ‘A’ and ‘B’, which recognize holes ‘a’ and ‘b’, giving rise to a fibrin network. (b, c) Hydrodynamic shear forces and thrombin concentration modulate the structure of fibrin networks. (d) Platelet contraction stiffens clots

Secondary hemostasis refers to the series of molecular interactions, serine protease cleavage and zymogen activation events that lead ultimately to the activation of thrombin protease, generation of a thrombin burst, and the formation of an insoluble fibrin clot. In the classical picture of the external pathway, tissue factor (TF) is released from endothelial cells upon membrane damage and binds to FVII to form an activated TF-FVIIa complex during an initiation phase (Hoffman 2003; Grover and Mackman 2018). This signal is then propagated by activating FIX (alternate pathway), FX, and FXI during the amplification phase. FXa then forms a prothrombinase complex with FVa which together activate thrombin (i.e., activated factor II (FIIa)). In addition to cleaving fibrinopeptides A and B (FpA and FpB) from fibrinogen (see below), thrombin also activates several of the upstream molecules in the cascade including FV, FVIII, the downstream transglutaminase FXIII as well as platelets, contributing to the feedback and amplification mechanism (Hoffman and Monroe 2001). An intrinsic pathway can also generate thrombin through signaling involving kallikrein protease, FXII and FXI; however, it is now thought to play a comparatively minor role in hemostasis and be more significant in inflammation and immunity (Long et al. 2016; Grover and Mackman 2019). The main role of thrombin is the activation of soluble fibrinogen into insoluble fibrin. Fibrin polymerization at the wound site then allows the formation of a fibrin network which exhibits viscoelastic properties essential for its function (Fig. 6). In the next sections, we outline what is known about fibrin assembly and structure, and how these structural features are related to its functional role in resisting fluid flow.

### Fibrin(ogen) structure and assembly

Fibrinogen is a ~ 340–420 kDa dimeric glycoprotein precursor to fibrin synthesized in the liver (Fig. 2b). Each subunit of the fibrinogen dimer contains three chains known as the Aα, Bβ, and γ chains(Chung et al. 1990). Within the fibrin(ogen) molecule, the six chains are held together by multiple disulfide bonds and oriented with their N-termini toward the central E region(Medved et al. 2009). From either side of the central nodule, the three chains extend into triple coiled-coil α-helix structures, terminating with a series of disulfide bonds that link the three chains at the C-ter ends of the coiled-coil connectors. Beyond these disulfide linkages, the C-terminal regions of the Bβ and γ chains fold independently to form the compact, globular β- and γ-nodules (D regions). The C-terminal portion of the Aα chain is different, forming a fourth α-helix and possessing an intrinsically disordered region which has up to now been uncrystallizable (Zhang and Redman 1994; Spraggon et al. 1997; Kollman et al. 2009).

Alternative splicing of the fibrinogen chain transcript results in the fibrinogen-420 (Fib-420) isoform, so named because of its higher molecular weight of 420 kDa (Fu et al. 1992; Fu and Grieninger 1994; Mosesson et al. 2004). Fib-420 makes up approximately one percent of circulating fibrinogen in adults; however, in neonate and infant populations it is upregulated 3–fourfold to concentrations of ~ 1 µM (Grieninger et al. 1997). Fib-420 differs from standard 340 kDa fibrinogen by replacement of each Aα chain by an AE chain splicing variant, containing an additional globular C-terminal extension domain (EC) encoded by splicing of the transcript to include exon VI.

The physiological function of Fib-420, more specifically the EC-domain, in infant and neonatal coagulation is unknown. The gene encoding exon VI found in the Fib-420 isoform is highly conserved among vertebrates(Fu et al. 1995). The EC-domain is homologous with the C-terminal regions of the B and γ chains found in the fibrin D region, but lacks a functional fibrin knob polymerization pocket. Remarkably, infant Fib-420 is found to assemble in a symmetric way, where the fibrinogen quaternary protein preferentially incorporates two copies of the same alternatively spliced AE chains. Few to no heterogeneously assembled fibrinogen molecules are found to contain one infantile AE chain and one conventional Aα chain(Fu et al. 1998; Applegate et al. 2000). The quality control system at work that regulates homogeneous assembly of Fib-420 into symmetric dimers is unknown. The EC-domain contains a binding site for 2 integrins involved in leukocyte adhesion and inflammation (Lishko et al. 2001) and has been reported to show chaperone-like activity (Tang et al. 2009). Fib-420 appears to have a slower rate of fibrin polymerization and lower turbidity than Fib-340 (Mosesson et al. 2004; Nellenbach et al. 2020). Electron microscopy and imaging has shown more highly branched and thinner fibers for Fib-420.

Another fibrinogen splicing variant is the gamma prime (γ’) variant (Henschen and Edman 1972; Mosesson et al. 1972; Mosher and Blout 1973; Chung and Davie 1984). The cDNAs of γ and γ’ chains share a common sequence encoding 1–407 amino acid residues; however, the γ chain contains an additional four amino acids (residues 408–411) at its C terminus, whereas the γ' chain contains 20 amino acids at its C terminus (residues 408–427). Therefore, the gene for the γ chain can be spliced to produce mRNAs that encode polypeptides with different C terminal sequences. Both of these polypeptides are integrated into fibrinogen present in plasma. These sequence differences between the γ chain and γ' chain explain the size and charge heterogeneity of fibrinogen.

The extreme C-terminal region of Aα chain, called αC region, is grouped into two sub-domains: the αC-connector and the αC-domain. The αC-connector consists of 10, 13-amino acid repeats and is thought to be unstructured. Circular dichroism (CD) and nuclear magnetic resonance (NMR) studies found that the lone disulfide bond in the αC-domain stabilizes a double β hairpin (Veklich et al. 1993; Tsurupa et al. 2009). In fibrinogen, the αC-domains are thought to interact with each other and with FpB in the E region (Litvinov et al. 2007). FpB (15 amino acids long) along with FpA (16 amino acids long) also located in the E region are cleaved by thrombin to form fibrin (Furlan et al. 1976).

Fibrinopeptide cleavage by thrombin exposes peptide sequences known as knobs ‘A’ and ‘B,’ which noncovalently bind the so-called hole regions ‘a’ (located in γ-nodules) and ‘b’ (located in β-nodules) (Fig. 6a). Initial fiber self-assembly is driven predominantly by knob ‘A’ interaction with complementary hole ‘a,’ while lateral fiber aggregation and later stabilization of formed clots are driven by knob ‘B’ interaction with complementary hole ‘b’ (Medved and Nieuwenhuizen 2003).

The knob/hole interactions between fibrin molecules allow the formation of double-stranded protofibrils of ~ 600 to 800 nm in length (Weisel et al. 1987). These protofibrils undergo lateral aggregation facilitated by αC-domains interactions both within and between protofibrils, leading to fibrin fiber formation and to a three-dimensional hydrogel (Weisel and Litvinov 2017). αC-domains interactions and the adjacent γ-nodules in protofibrils are further reinforced through cross-linking by the plasma transglutaminase Factor XIIIa (FXIIIa) **(**Fig. 2b). Prior to FXIIIa crosslinking, fibrin assembly is reversible (Chernysh et al. 2012) while after crosslinking the clot is irreversibly cross-linked and found to be stiffer and more resistant to fibrinolysis (Collet et al. 2005).

### Viscoelastic properties of fibrin networks

Fibrin is a viscoelastic polymer, sharing mechanical properties with both elastic solids and viscous liquids. These viscoelastic properties are physiologically important because of fibrin’s role in performing the mechanical task of stemming blood flow (Fig.6). The elastic component contributes to in-phase mechanical resistance of the network under reversible mechanical deformation, while the viscous component contributes to out-of-phase mechanical resistance in proportion to the strain (loading) rate (Weisel and Litvinov 2017). The stress–strain curve for fibrin shows that stress is directly proportional to strain at low strain values, with constant slope until a strain of ~ 80% is reached. At larger strains, the slope of the curve then increases dramatically. This nonlinearity, referred to as strain hardening or strain stiffening, is an interesting property of select protein-based hydrogels (Weisel and Litvinov 2017). Strain-stiffening is thought to originate from structural hierarchy at the molecular level. These models imply that fibrin deformation is accompanied by structural rearrangements at multiple different length scales.

At the clot scale (mm), stretching a fibrin gel is accompanied by dramatic shrinkage due to water expulsion and network densification. Fibrin is one of the most extensible polymers, and under stress blood clots can be severely stretched without breaking. For example, it was reported that fibrin fibers and plasma clots stabilized with Factor XIIIa could be stretched to over three times their relaxed length before breaking (Liu et al. 2006; Brown et al. 2009). At the fiber scale (µm), fibers align along the strain direction (Fig. 6b) (Brown et al. 2009; Litvinov et al. 2012) becoming thinner and bundled (Brown et al. 2009). In response to shear stress, fibrin fibers buckle and bend in response to the direction of flow (Lindström et al. 2013). The elastic limit of a fibrin fiber was tested by stretching with an atomic force microscope (AFM) tip. After being stretched to 2.8 times its original length, the crossed-linked fiber recovered without permanent damage (Liu et al. 2006). Finally at the molecular scale (nm), reversible unfolding of the central coiled-coil connectors (Brown et al. 2009; Hudson et al. 2013) and stretching of αC region of fibrin can contribute to work dissipation (Collet et al. 2005; Duval et al. 2014). Work dissipation refers to the mechanical energy that must be input in order to unfold and stretch fibrin, and these sacrificial structures in fibrin are thought to be essential for fibrin’s viscoelastic properties.

### Fibrinolysis

To avoid over clotting, fibrin is degraded through a process called fibrinolysis mediated by the enzyme plasmin (Pln), a multi-domain serine protease (Fig. 2c). Derived from the zymogen precursor plasminogen (Plg), Pln is the major fibrinolytic protease, circulating in the bloodstream at a concentration of ∼2 µM (Collen et al. 1972). Pln is a promiscuous trypsin-like protease that cleaves peptides C-terminal from lysine and arginine residues in many targets. Pln activates other proteases and growth factors involved in inflammation, cell migration, angiogenesis, and tumor growth among others (Weisel and Litvinov 2017).

Native circulating Plg (92 kDa) is a single chain glycoprotein, cross-linked by 24 disulfide bridges. It contains an N-terminal activation peptide (NTP), five homologous triple loop kringle domains (K1–K5), and a serine protease domain (Fig. 2c) (Forsgren et al. 1987; Law et al. 2012). K1, K2, K4, and K5 contain a DXD/E motif that bonds C-terminal lysine residues, which is likely a primary binding mechanism of plasmin(ogen) to fibrin and/or cell surfaces (Castellino and Ploplis 2005). Thus, as fibrin is digested, newly generated C-terminal lysine residues on the digested fragments become exposed, providing more binding sites for Plg and creating a positive feedback and amplification mechanism that accelerates degradation.

Plg can be converted to Pln by both tissue-type Plg activator (tPA) as well as by urokinase-type activator (uPA), although tPA is thought to be the predominant Plg activator during fibrinolysis (Hudson 2017). Both tPA and uPA are multidomain serine proteases. While uPA binds to a cell-surface receptor (urokinase-type plasminogen activator receptor, (uPAR)), tPA binds directly to fibrin through the Kringle domain or through a finger domain (Hudson 2017). tPA and uPA cleave the Arg561–Val562 bond of Plg, producing two-chain active Pln with an N-terminal heavy chain (12–65 kDa) and a C-terminal light chain (25 kDa). Once activated, Pln can cleave both tPA and uPA, converting them from single chains to activated two-chain polypeptides (Cesarman-Maus and Hajjar 2005).

Plg and tPA both bind to fibrin, thereby localizing and enhancing Pln generation in fibrin substrates. In the absence of fibrin, tPA is a weak activator of Plg, but its catalytic efficiency is enhanced > 100 fold in the presence of fibrin (Cesarman-Maus and Hajjar 2005). The affinity between tPA and Plg also increases in the presence of fibrin. Fibrin (but not soluble fibrinogen) was found to enhance the activation of Plg by tPA (Rijken et al. 1982). One active hypothesis is that cleavage of fibrinogen to produce fibrin networks causes a structural change that generates new conformational epitopes for Plg and tPA binding (Medved and Nieuwenhuizen 2003).

The structure and density of fibrin fibers can significantly influence the enzymatic digestion rate. Fibrin network structure is influenced by fibrinogen, thrombin, and ion concentrations including Ca^2+^ and Zn^2+^ (Ryan et al. 1999; Xia et al. 2021). High fibrinogen or thrombin concentrations, and high ionic strength produce thinner fibers with higher density (Ryan et al. 1999), while high Ca^2+^ (while keeping ionic strength constant by adjusting NaCl concentration) or low thrombin concentrations produce clots with thicker fibers and lower density (Fig. 6c). It was reported that thick fibers lyse more slowly than thin fibers (Collet et al. 2000; Bannish et al. 2014; Bucay et al. 2015) and that low density networks of thick fibers lyse more rapidly than high density networks composed of thin fibers (Carr and Alving 1995; Liu et al. 2010; Machlus et al. 2011). However, there have been contradictory reports (Gabriel et al. 1992; Kolev et al. 1997), with recent modeling studies seeking to reconcile the differences by accounting for the number of tPA molecules able to initiate lysis (Hudson 2017).

Another parameter that influences fibrinolysis efficiency is mechanical force. Varjù and colleagues showed that mechanical stress renders fibrin clots more resistant to fibrinolysis (Varjú et al. 2011), an observation with two origins. Firstly, activation of Plg by tPA was decreased two- to threefold in contact with stretched fibrin fibers as compared to relaxed ones. Secondly, once Pln is formed, it is less active in the presence of strained fibrin. Stretched fibers were lysed 50% less by tPA/Pln as compared to unstretched fibers. In more recent studies, two teams worked on the lysis susceptibility of single fibrin fibers (Bucay et al. 2015; Li et al. 2017). Using different experimental set ups, both studies confirmed at the individual fiber scale the same trend that was observed by Varjù at the clot scale: that fibrinolytic activity is dependent on the degree of strain experienced by fibrin fibers, even at high plasmin concentrations.

It was also demonstrated in several studies that retracted clots are more resistant to fibrinolysis (Longstaff et al. 2011; Whyte et al. 2017). Activated platelets generate contractile forces that propagate through the fibrin network (Lam et al. 2011), leading to clot retraction, shrinkage and expulsion of fluid. This process is important for stabilizing clots through strain stiffening and for maintaining unobstructed vessels. One mechanistic explanation is that fiber alignment due to shear stress or clot retraction by platelets induces a reduction in pore size and a densification of tPA and Pln binding sites, lowering the mass transport rate of fibrinolytic agents through the material (Adhikari et al. 2012; Tutwiler et al. 2018). Clot lysis rates therefore depend on several factors, including accessibility of fibers to fibrinolytic enzymes, pore sizes, and mechanical strain (Cone et al. 2020). In Fig. 2, we have provided protein structural renderings of some of the key molecules covered thus far.

## Single-molecule experiments on coagulation proteins

Mechanical forces acting on folded domains or ligand–receptor interactions are an integral part of many biological processes, and they are especially relevant to consider for proteins involved in hemostasis and coagulation. In the last twenty years, single-molecule mechanical experiments have allowed molecular scale studies on the physiological function of these proteins to decipher the mechanisms of activity regulation by mechanical force. Some of the relevant molecular mechanisms regulated by mechanical forces include protein interaction stabilization (i.e., catch bonds), exposure of cryptic binding sites or scissiles bonds, force-dependent unfolding and refolding events, and protein structural changes that trigger biochemical signaling cascades (Vogel 2006; Puchner and Gaub 2012).

### VWF unfolding and ADAMTS-13 cleavage

The first single-molecule study on force regulated activation of VWF was carried out using optical tweezers (OT) on the single A2 domain (Zhang et al. 2009). It was already known that the cleavage of the A2 domain was activated by shear in large VWF concatemers. It was thought that tensile force altered the conformation of A2 domains to enable cleavage and that the scissile bond was likely buried in the native state. Therefore, in this study they tested whether unfolding of the A2 domain occurred at physiological forces experienced by VWF in the circulation, and whether mechanical unfolding could prime A2 for cleavage by ADAMTS-13. A single A2 domain coupled to DNA handles through N-ter and C-ter Cys tags was stretched using a bead-based laser trap and micropipette. The A2 domain was subjected to mechanical unfolding/refolding and force clamp cycles. A2 was found to unfold at tensile forces of 7–14 pN at loading rates of 0.35–350 pN・s^−1^ and could refold in the absence of force with a rate of 0.54 ± 0.05 s^−1^. They discovered that mechanical unfolding of A2 was indeed required for cleavage by ADAMTS-13.

Two years later, the high-resolution A2 crystal structure helped to confirm the presence of a Ca^2+^ binding sites with micromolar affinity for this partner (Xu and Springer 2012). It consists of a water molecule and five coordinating residues located upstream of the scissile strand (Zhou et al. 2011a; Jakobi et al. 2011). This metal ion plays a crucial role in A2’s sensitivity to ADAMTS-13. In the absence of Ca^2+^, ADAMTS-13 cleavage is more efficient than in the presence of the ion. Ca^2+^ binding prevents A2 unfolding and therefore, provides protection from proteolysis in the absence of shear stress (Zhou et al. 2011a; Jakobi et al. 2011; Xu and Springer 2012). Using OT, it was shown that Ca^2+^ binding does not significantly affect A2 unfolding; however, it has a large impact on the domain refolding kinetics, increasing several fold the folding rate constant (Jakobi et al. 2011; Xu and Springer 2012).

VWF being a multi-domain protein, other studies have also been carried out on larger constructs, to place A2 in a more physiologically relevant environment. Ying and colleagues performed unfolding experiments on VWF multimers purified from human plasma along with a polyprotein (A1-A2-A3)_3_ construct, using OT (Ying et al. 2010). Proteins were immobilized on latex beads functionalized with specific antibodies. In the resulting data, extensional jumps signifying domain unfolding were observed, and unfolding forces and contour length plots showed two or three peaks with integer multiples of ~ 21 pN and ~ 63 nm, respectively. These integer multiples indicated unfolding of single identical domains, likely A2, in parallel. Stretching of minimal (A1-A2-A3)_3_ polyproteins also produced comparable distributions, showing that A2 unfolding accounts for the behavior of the VWF multimers.

Until recently, the exact nature of the interactions holding VWF in the compact conformation was not well understood. In 2016, two AFM studies on VWF dimers linked “tail to tail” through CTCK disulfide bridges (corresponding to the smallest repeating subunits of VWF multimers) were reported where Müller and colleagues identified strong intra-dimer interactions (Müller et al. 2016b, 2016a). These studies identified strong inter-monomer interactions involving the D4 domain and reported a dependence of binding strength on divalent ions and pH. Variations in VWF multimer compactness and stability may play a key role during VWF processing, multimerization, and storage occurring in ER (pH 7.4), Golgi (pH 6.8) and Weibel–Palade bodies (pH 5.4). It is also noteworthy that VWF has the highest mechanical strength at physiological pH. The mechanostability decreases at lower pH values, a feature that is physiologically relevant due to the acidification that can occur in connection with injury and inflammation, although the timescale of acidification remains unclear (De Backer 2003; Christou et al. 2005).

Recently, the use of magnetic tweezers (MT) allowed researchers to unravel another structural transition occurring at the dimer scale of VWF (Löf et al. 2019). MT is a useful tool because of the ability to apply extremely low forces to molecules in the range of ∼0.01–100 pN, and to measure many molecules in parallel (Lipfert et al. 2009). This study carried out in the low force regime showed that the first step of VWF mechano-activation is unzipping of the C stem domains interacting together in the context of the dimer (Fig. 1b). Previous AFM imaging and electron microscopy studies suggested that the VWF stem consisting of the 6 C domains behaves in a zipper-like fashion; however, direct observation of the transitions of the stem had not been previously reported (Zhou et al. 2011b; Müller et al. 2016b, 2016a). At only ∼1 pN of force, the VWF stem region showed reversible zipping/unzipping transitions with a contour length increase of ∼50 nm. The stem transitions did not behave as a 2-state system, but showed more complex multistate behavior which was consistent with AFM observations, showing that the stem frequently populates partially open configurations (Müller et al. 2016b, 2016a). In these experiments, they also recorded A2 unfolding events and intermonomer interaction through D4 domains. They found opening of the intermonomer interactions mediated by D4 occurred in the same force range as A2 unfolding, suggesting both behaviors could help regulate VWF’s hemostatic activity under shear stress in the bloodstream (Löf et al. 2019). This consequential work was also the occasion to study the influence of A2 Ca^2+^ binding in the context of the VWF dimer using a more sensitive technique and more precise experimental setup. Löf and colleagues found that at different constant forces, unfolding rates were 2- to 4-times higher in EDTA buffer and 2-times slower in Ca^2+^ containing buffer compared to previous studies using OT on isolated A2 domains (Jakobi et al. 2011; Xu and Springer 2012). Since no signal could be attributed to dissociation of A2 with neighboring domains, the authors could not conclude a shielding effect of A2 surroundings. Instead, they suggested that these substantial differences are coming from the use of the Dudko–Hummer–Szabo method to transform the rupture force distribution, this method being sensitive to the elastic response of the DNA handles that were used for the OT studies. Löf and colleagues’ MT experimental design involved a more recently developed sortase A and YbbR tag immobilization technique (Yang et al. 2020), allowing single-molecule stretching without the need for DNA handles. As for the refolding rates, their results were in good agreement with OT experiments: they were two- to sixfold higher in the presence of Ca^2+^ than with EDTA at forces between 2 and 5 pN. This value reached a factor of 20 difference in the absence of applied force, indicating a significant stabilizing effect of the metal ion (Löf et al. 2019). Overall, Ca^2+^ would both decrease the unfolding and increase the folding rates of the A2 domain making this cation a major contributor of VWF mechanostability and therefore, a major regulator of hemostasis.

### GpIb-A1 interaction

Hydrodynamic forces play a role not only in A2 domain unfolding and protease cleavage, but also in VWF activation through exposure of the A1 domain that promotes platelet arrest at the injury site. It was recently shown that the flanking sequences of the A1 domain (about 30 residues each) form a so-called discontinuous autoinhibitory module (AIM) and play an inhibitory role by masking the GpIbα binding site (Deng et al. 2017, 2018). OT experiments helped to determine the mechanical properties of this AIM (Arce et al. 2021). A recombinant AIM-A1 protein with a N-ter biotin and a C-ter SpyTag was fixed to a SpyCatcher-biotin DNA handle, held in an OT, and repeatedly extended and relaxed. The authors demonstrated that the AIM behaves as a single structural unit, unfolding between 10 and 20 pN most frequently in a single step, as opposed to separate unfolding steps for the N-ter AIM and the C-ter AIM.

The interaction mechanism between the platelet receptor GpIbα and A1 domain was also shown to be force-resistant using single-molecule techniques (Kim et al. 2010). Kim and colleagues developed a new single-molecule assay for repeated measurements of receptor-ligand binding/unbinding called ReaLiSM. The C-ter of the A1 domain (a construct that did not include the AIM) was fused to the N-ter of GpIbα with a 43-residue linker. DNA handles were attached to cysteine residues at the C-ter of GpIbα and N-ter of A1. The construct was held through the handles in a micropipette and bead-based laser trap, allowing them to subject the A1-GpIbα construct to cycles of mechanical force. At low loading rates, bond ruptures generated a narrow unimodal force distribution, while at higher pulling rates, the distribution of dissociation forces was bimodal. They developed a kinetic model to explain the two bond dissociation pathways: the first dissociation pathway predominated below 8 pN, whereas above 12 pN the second pathway predominated. This second state has a ∼20-fold longer lifetime due to a lower off-rate that can potentially resist hydrodynamic forces that could disaggregate the platelet plug. They found that tensile force stabilizes the high-affinity state and strengthens the interaction under shear stress. They concluded that the A1-GpIbα bond is a flex-bond, with a low stability state transitioning to the second high stability state upon mechanical loading (Fig. 4). The two bond states were observed not only for unbinding, but also for rebinding, suggesting the existence of two different conformational states of A1 that are present prior to binding to GpIbα (Kim et al. 2010, 2015). This hypothesis was already formulated by previous denaturation and structural studies of A1 domain and mutants (Auton et al. 2007; Tischer et al. 2014) and was recently confirmed by Fu and colleagues *(*Fu et al. 2017*)*.

### Platelet mechano-sensing

Platelets sense mechanical shear forces and transduce them into biochemical signals to promote clot growth and stabilization. GpIbα together with GpIbβ and GpIX forms the GpIb-IX trans-membrane protein complex (Fig. 5). In frame with the leucine-rich repeat domain (corresponding to the ligand binding domain, LBD) of the N-ter, GpIbα comprises a glycosylated macroglycopeptide region, a stalk region, a pair of cysteine residues form disulfide bridges to GpIbβ, a transmembrane helix, and a short C-terminal cytoplasmic domain (Qiu et al. 2015).

Zhang and colleagues showed in a single-molecule study that the stalk region can unfold under tension and act as a mechanosensing domain (Fig. 5) (Zhang et al. 2015). In the OT experiment, recombinant VWF-A1 was attached through a DNA handle to a trapped bead. Biotinylated GpIb-IX was held on a streptavidin bead fixed with a micropipette. In the pulling curves, an unfolding event could be observed before the rupture of the complex, with forces ranging from 5 to 20 pN. Worm-like chain (WLC) fitting of these data suggested the unfolding of a domain of ∼63 residues that was referred to as the Mechano-Sensitive Domain (MSD). Using antibodies targeting different regions of the GpIb-IX receptor allowed them to localize the MSD in the stalk region of GpIbα chain (Ibα-S). The MSD unfolded at forces similar to those required to induce flex-bond formation between VWF-A1 and the N-ter domain of GpIbα. Another recent OT study was carried out by Zhang and colleagues using a monoclonal antibody recognizing the macroglycopeptide region of GpIbα (Zhang et al. 2019). This allowed them to record pulling and relaxation cycles and led to the first direct detection of refolding of the MSD in GpIb-IX.

### Fibrin(ogen)

Several single-molecule studies have characterized the molecular transitions that fibrin(ogen) undergoes when exposed to mechanical stress. These results provide single-molecule evidence and explanations for the extraordinary viscoelastic properties of fibrin networks. It was shown very early that structural changes during fibrin deformation under mechanical stress include unfolding of the central coiled-coil connectors. The first AFM pulling experiments on fibrinogen (Brown et al. 2007) reported covalent cross-linking of fibrinogen molecules with FXIIIa and direct application of this solution of oligomerized fibrinogen onto a mica surface. When unfolded under force with an atomic force microscope cantilever tip, fibrinogen oligomers generated periodic sawtooth patterns. Shortly afterward, AFM and steered molecular dynamic (SMD) simulations were performed on single fibrinogen molecules and fibrin protofibrils (Lim et al. 2008). The molecules were directly adsorbed to a coverslip surface, leading to extremely low success rate for picking up single molecules (approximately 1% over all trials). Pulling single fibrinogen molecules produced force extension curves characterized by three phases, a behavior already characterized previously for coiled-coil helical structures such as myosin coiled-coil (Root et al. 2006), leucine zipper coiled-coils (Bornschlögl and Rief 2006), desmin intermediate filaments (Kiss et al. 2006), and even DNA (Rief et al. 1999). These three unfolding phases were attributed to specific structural features undergoing mechanical denaturation and unfolding with interpretations supported by molecular dynamics simulations.

It was later found using Fourier transform infrared (FTIR) and Raman spectroscopy that the extension of the oligomers is accompanied by the transition of the triple α-helix to β-sheet (Litvinov et al. 2012; Fleissner et al. 2016). This structural transition was further analyzed using all-atom molecular dynamics simulations of fibrin(ogen) unfolding (Zhmurov et al. 2012). A more recent AFM study and pulling simulations of single fibrinogen molecules and oligomers helped refine the unfolding mechanism (Zhmurov et al. 2011). They showed that forced unfolding is a collective process involving mechanically coupled elements. They proposed that the α-helical coiled-coil connectors in fibrin(ogen) act as mechanical capacitors where mechanical energy can be reversibly stored through unfolding and refolding transitions. This AFM study also confirmed that the extension of fibrin(ogen) is largely determined by unfolding transitions of the C-ter γ-nodules (Averett et al. 2008, 2009).

An essential aspect of protofibril and fiber structures was not taken into account in these early studies, which is the role of αC-domain. FXIIIa ligation of Aα chains contributes substantially to fibrin clot stiffness and elasticity (Collet et al. 2005; Duval et al. 2014). At strains below 100%, αC regions play a predominant role in the extension and recoil of the fibers due to the long-unstructured region upstream of αC β-hairpin domain. This region would act as an entropic spring allowing fibrin fibers under low stress to recoil within milliseconds (Hudson et al. 2013).

Fibrin knob/hole interactions also play a role in the mechanical response of fibrin clots to mechanical forces, in particular the knob ‘A’ interaction with complementary hole ‘a’ which was reported to be a force-activated catch bond. Litvinov and colleagues reported a single fibrinogen- or fragment D-coated bead that was trapped and repeatedly brought into contact with a fibrin or fragment E-coated pedestal using OT (Litvinov et al. 2018). The strength of the A:a knob/hole interaction was found to increase with tensile force up to f ≈ 30–35 pN and then, decrease at f > 35 pN. They identified a movable flap corresponding to residues γ295 to γ305 forming a short α-helix, which regulates the opening/closing of the binding interface in response to force. Under low force, the movable flap interacts with residues in the γ-nodule, leaving the binding interface open while under tension, it extends and stabilizes the high affinity bound state.

## Biophysical properties of fibrin networks

The biophysical performance of fibrin networks is strongly influenced by factors such as clotting factor concentrations, ionic strength, platelet contraction, and fluid flow conditions, among several others (Jansen et al. 2013; Kurniawan et al. 2014; Badiei et al. 2015; Mihalko and Brown 2020). Deviation from standard physiological concentrations of clotting factors is associated with a number of pathologies in which fibrin networks are formed with substandard biophysical and mechanical properties, resulting in insufficient hemostatic ability. In this section, we outline how natural clotting factors and fibrin-binding synthetic molecules can influence fibrin network properties, including mechanical strength, fiber thickness, density, degree of branching, porosity, and resistance to degradation by plasmin. We discuss the implications of these effects on the ability of blood clots to achieve adequate hemostasis.

### Endogenous mediators of fibrin network structure

#### Thrombin

Thrombin is a multi-functional serine protease that is activated at the downstream end of the clotting cascade and initiates fibrin gelation by cleaving the N-termini of Aɑ and Bβ polypeptides in the central E region of fibrinogen (Weisel and Litvinov 2017). The concentration of thrombin is known to strongly influence clot structure. Clots formed at low thrombin concentrations have thicker, less dense fibers and are prone to rapid fibrinolysis, while those formed at high thrombin concentrations have thinner, denser fibers that are more resistant to proteolytic degradation (Wolberg 2007)*. *In vitro assays to test the effects of thrombin concentration on clot structure typically involve adding a fixed amount of activated thrombin to a fibrinogen solution. Although this approach is straightforward, it may not reflect the dynamic nature of thrombin generation in vivo, where free thrombin concentrations can range from 1–500 nM during coagulation depending on environmental conditions (Wolberg 2007). Environmental conditions can also modulate the activity of thrombin to change clot morphology. For example, Ca^2+^ is required for the assembly of procoagulant complexes and for the generation of thrombin, and clotting onset times are shorter in the presence of Ca^2+^, resulting in clots with thicker fibers than clots formed in the presence of thrombin alone (Carr et al. 1986).

The mechanism by which varying thrombin concentration and/or activation rates drive changes in fibrin network structures is related to the varying rate of fibrinopeptide release, which in turn influences the rate of protofibril formation and lateral aggregation (Wolberg and Campbell 2008). Higher thrombin concentrations are associated with fast fibrin monomer activation and protofibril extension, which may account for the reduced lateral aggregation of protofibrils that was observed by Domingues et al*.* Using a number of non-destructive techniques including turbidimetry and AFM, the researchers found that increasing thrombin concentrations led to a significant decrease in the average number of fibrin protofibrils per fiber, but only a minor reduction in the size of those fibers, indicating that compaction of protofibrils, was significantly reduced under these conditions (Domingues et al. 2016). This result was in contrast to previous results gathered from SEM studies, which showed a significant reduction in fiber diameter in the presence of higher thrombin concentrations (Wolberg 2007). This discrepancy was attributed to the differences in solvent content within fibers formed at low vs. high thrombin concentrations; fibers formed at low thrombin concentrations had higher protein density and less intrafibrillar solvent content, and so contracted less during the dehydration step of SEM sample preparation, causing them to appear thicker in SEM images (Domingues et al. 2016).

### Dynamic stiffening through contractile stress generated by cells

Platelets and fibroblasts bind fibrin networks and alter their structural and mechanical properties by exerting stresses through cytoskeletal contraction. Platelet plugs are supported by the fibrin network that forms during secondary hemostasis. Fibroblasts are recruited into a clot from the surrounding ECM during the proliferation phase of wound healing, and work to promote the formation of new ECM at the site of injury through the release of collagen, fibronectin, and proteoglycans (Chester and Brown 2017). Fibrin-bound platelets decrease clot sizes by increasing fiber density which increases the elastic modulus as much as tenfold through actomyosin-based contraction of their cytoskeletal network (Lam et al. 2011; Wufsus et al. 2015). In a landmark study, Lam et al. used atomic-force microscopy (AFM) to investigate the contractile forces generated by individual platelets bound to fibrinogen-coated cantilevers and surfaces. They found that each individual platelet can exert a contractile force of up to 29 nN, and that platelets adhered to fibrinogen at forces up to 70 nN. Based on these results, they proposed a mechanism of platelet-mediated contraction of fibrin, whereby platelets exert higher contractile forces on regions of higher fiber density, such that contraction of the clot as a whole is more uniform and the overall elasticity of the clot is increased (Fig. 6) (Lam et al. 2011). A subsequent work by Kim et al*.* elucidated the structural mechanism of platelet contraction at the single cell level. This mechanism involved the extension and retraction of the filopodia of platelets that were bound to fibrin fibers, the end result of which was the shortening and bending of those fibers and the introduction of kinks in the fiber network. This so-called hand-over-hand action of filopodia pulls on fibers transversely to their longitudinal axis, leading to an increase in fiber density (Kim et al. 2017).

Fibroblasts migrate into the clot during the proliferation stage of wound healing following the release of fibroblast growth factor (FGF) from macrophages, and their key functions are then regulated through the secretion of TGF-β from macrophages, platelets, and lymphocytes (Chester and Brown 2017). Through their binding to fibrin, fibroblasts like platelets can exert contractile forces on fibrin networks in a manner that is theorized to relate to the nonlinear elasticity of those networks. Winer et al*.* proposed this theory after conducting AFM and rheological experiments on fibroblasts embedded within fibrin gels. They found that embedded fibroblasts reached maximal spreading even in fibrin networks with low elastic moduli, in contrast to their behavior in low stiffness linearly elastic gels, where they maintain a more rounded morphology (Winer et al. 2009). Jansen et al*.* investigated this idea further using a similar model of fibroblasts embedded in fibrin gels and found that fibroblasts exert myosin-II-driven contractile forces on fibrin networks while spreading, which led to local alignment of fibrin fibers around cells. They proposed that these forces push the surrounding fibrin gel into the nonlinear viscoelastic region and thereby drive strain-stiffening of these materials (Jansen et al. 2013; Litvinov and Weisel 2017).

### Factor XIII

Factor XIII is a clotting-associated transglutaminase that exists in vivo as a tetrameric zymogen prior to conversion into an active dimer (FXIIIa) through the action of thrombin. FXIIIa catalyzes an acyl transfer reaction between lysine and glutamine on the Aɑ and γ chains of fibrin. The resulting covalent cross-links increase fibrin’s mechanical stability and resistance to proteolysis (Bagoly et al. 2012). Inherited deficiencies in FXIIIa functionality, though rare, invariably lead to severe and persistent bleeding disorders (Muszbek and Katona 2016).

The improvements in clot properties that are observed in the presence of FXIIIa are believed to originate from structural changes to fibrin networks. Hethershaw et al*.* conducted turbidity measurements on fibrin clots formed with or without FXIII to elucidate what effect the presence of FXIII had on fibrin fiber thickness and density (Hethershaw et al. 2014). Turbidity assays are established for investigating structural properties of fibrin, and networks formed from thinner, more densely packed fibers tend to be less optically dense than those formed from larger diameter, loosely packed fibers (Sproul et al. 2018). The researchers found that clots formed in the presence of FXIII had significantly lower turbidity than those formed without FXIII. Follow-up SEM studies confirmed that fiber diameter was significantly smaller (by approx. 10 nm) and density significantly higher (by approx. 0.6 fibers/µm) for clots formed with FXIII.

Kurniawan and colleagues also applied turbidity assays to study fibrin gelation with and without inhibition of FXIII. They found that there was little effect of FXIII inhibition on the initial phase of clot formation (approx. 10 min under their conditions) during which fibrin protofibrils are aggregating laterally, but that FXIII inhibition did lead to the elimination of the secondary phase of gelation (lasting several hours), during which compaction of the fibrin network occurs (Kurniawan et al. 2014). Subsequent rheological studies showed that FXIII significantly increased the linear elastic modulus of clots, but that it did not affect the stiffness of these clots at high strains. Given these results, the researchers proposed a multiscale structural model of fibrin networks, whereby FXIIIa cross-links and compacts protofibrils within a fiber. Thermal fluctuations are believed to dominate the elastic response of the clots at low-strains, while at higher strains the stretching of individual protofibrils dominates the elastic response. Based on this model of fibrin elasticity, FXIIIa-mediated compaction is critical at low strains but plays less of a role at high strains.

### Hydrodynamic shear stress

In vivo blood clots are exposed to hydrodynamic shear from physiological flow of blood through veins (10–100 s^−1^) and arteries (500–1500 s^−1^), and during extravasation of blood from vessels following injury (Campbell et al. 2010). The physical stimulation of fluid flow is known to have a strong influence on the structure and mechanical properties of fibrin networks. Campbell et al*.* investigated this by studying the formation of clots from platelet-free plasma (PFP) in the presence of immortalized human dermal fibroblasts (NHF_1_-hTert) under static and flow conditions. They found that clots formed under hydrodynamic flow had significantly larger, and more densely-packed fibers than clots formed under static conditions (Campbell et al. 2010). This can be explained by the fact that, for a given blood concentration of fibrin, a greater amount of fibrin will be deposited on a forming clot under flow than under static conditions due to constant replenishment of the reactant. Clots formed under conditions of flow therefore have higher protein content (Weisel and Litvinov 2017). Additionally, SEM studies showed that fibrin networks formed under flow had significantly higher degrees of anisotropy as compared to those formed under static conditions, which were largely isotropic.

Badiei and colleagues conducted controlled stress parallel superposition (CSPS) rheological experiments on incipient fibrin clots, whereby an oscillatory shear stress was superimposed on top of a steady state shear stress in order to study the effects of unidirectional flow/shear stress on the formation, fractal microstructure, and stiffness of fibrin. They found that clots formed under conditions of unidirectional shear stress exhibited greater network compaction, which was correlated with an increase in their shear storage moduli (G’) (Badiei et al. 2015). Based on this series of studies, it is clear that the physical stimulation of fluid flow can induce structural and functional changes in fibrin networks that influence the mechanical properties and performance of clots.

### Synthetic mediators of fibrin network structure

#### Synthetic platelets

A key function of platelets in coagulation is to bind and compact fibrin fibers through actin-myosin-mediated contraction of the cytoskeleton. Synthetic platelets or platelet substitutes seeking to recapitulate this function should meet two important criteria: (A) they should bind fibrin with high specificity while not binding to fibrinogen; and (B) they should promote compaction of fibrin fibers through the application of contractile forces or through aggregation. Brown et al*.* achieved such a system consisting of ultrasoft microgel particles coated with fibrin-binding nanobodies, which they called platelet-like particles (PLPs). Using a precipitation polymerization method, they synthesized ultra-low density crosslinked poly(N-isopropylacrylamide-co-acrylic acid) microgels and decorated them with variable domain-like recognition motifs (single-domain VHH antibodies or nanobodies) that had been selected in vitro by phage display biopanning to bind fibrin with high specificity (Brown et al. 2014; Welsch et al. 2018). Not only were PLPs able to promote the formation of fibrin clots in a manner akin to platelet-rich plasma (PRP), but upon binding to fibrin the ultra-soft particles would collapse, causing each PLP to exert a contractile force of approximately 6.5 ± 5.5 pN. While this is significantly lower than the contractile forces individual platelets were found to be able to exert (from 1.5 to 79 nN) (Lam et al. 2011), PLPs were still able to stiffen clots significantly and compact their local fibrin network. Ultimately, this resulted in clots that were more resistant to degradation, promoted wound healing, and reduced bleeding times in murine models of vascular injury (Brown et al. 2014; Nandi et al. 2019).

Other examples of synthetic platelet mimics include PLGA-PLL nanoparticles with surface exposed RGD motifs that are able to bind endogenous platelets, and lipid vesicle decorated with a number of platelet-associated peptides such as vWF and various integrins (Bertram et al. 2009; Dyer et al. 2018). Sekhon et al*.* recently described platelet-mimicking procoagulant nanoparticles (PPNs) which comprised liposomes displaying phosphatidylserines; this anionic phospholipid is exposed on platelets endogenously following their activation, and their presence on PPNs led to increased thrombin generation and improved hemostasis in animals treated with these particles (Sekhon et al. 2022).

### Fibrin-binding polymers

Synthetic polymers have also been designed with the ability to bind fibrin both through engagement of fibrin’s natural knob/hole polymerization mechanism, or orthogonally through the use of cyclic peptides with high binding affinity for fibrin. There have been several reports of polyethylene glycol (PEG) bearing knobs ‘A’ and ‘B’ peptide mimics that affect the structure of fibrin clots. A summary of how knobs ‘A’ and ‘B’ mimics and other fibrin network modulators affect fibrin-based biomaterials can be found in a review by Brown and Barker (Brown and Barker 2014). Stabenfeldt et al*.* introduced PEG functionalized with knob ‘B’ mimics to polymerizing fibrin gels. Engagement of hole ‘b’ by knob ‘B’-PEG led to the formation of fibrin networks with greater porosity, but also, surprisingly, increased mechanical stiffness and greater resistance to fibrinolysis (Stabenfeldt et al. 2012). Through this method, the researchers were able to improve the mechanical robustness of clots without sacrificing mass transport through the network, as evidenced by the ability of the modified clots to support angiogenesis at a level equivalent to control clots.

Another example of a synthetic fibrin-binding polymer was reported by Pun and co-workers. Their design consisted of a (hydroxyethyl)methacrylate (HEMA)- N-hydroxysuccinimide methacrylate (NHSMA) polymer backbone p(HEMA-co-NHSMA), which they functionalized with a cyclic peptide isolated by phage display with high affinity and specificity for fibrin (Chan et al. 2015a). When the functionalized polymer (polySTAT) was introduced into forming fibrin gels, it produced a number of structural changes in fibrin networks, including reduced porosity, fiber densification and increased mechanical stiffness as compared to clots formed in the presence of non-fibrin-binding polymers or FXIII. Follow-up neutron scattering studies indicated that polySTAT enhanced clot properties likely by increasing the radius of fibers through recruitment of additional fibrin to those fibers (Lamm et al. 2017). Ultimately, these improved clot characteristics translated to reduce bleeding times and improve survivability in animal models of hemorrhage.

Our research group has also recently described new formulations of polymers that modulate the properties of fibrin clots. In this case, our designs are based on a particular class of intrinsically disordered proteins called elastin-like polypeptides (ELPs). ELPs are repetitive pentapeptide sequences derived from the hydrophobic domains of human tropoelastin. ELPs exhibit stimuli-responsive solubility whereby they phase separate from aqueous solutions at temperatures above a lower critical solution temperature (LCST). The LCST can be tuned by modifying ELP properties such as length, concentration, and composition (Varanko et al. 2020). We recently reported hemostatic ELPs (hELPs) that were able to covalently bind fibrin (Urosev et al. 2020). The covalent cross-linking was achieved by interspersing contextual glutamine- or lysine-containing peptide sequences along the length of hELP. These sequences were recognized by FXIIIa such that when hELPs were mixed with a gelling fibrin clot, they were covalently integrated into fibrin networks by activated FXIII. The resulting hybrid hELP/fibrin clots exhibited improvements in mechanical strength, resistance to fluid flow, and resistance to plasmin degradation. Interestingly, improvements in clot mechanical properties were only seen when hELPs were integrated into clots at physiological temperature (i.e., above their transition temperature), indicating that phase separation of hELPs was a necessary factor in producing the stiffening effect. hELPs therefore offer a protein-based alternative to the previously described synthetic polymer hemostats, with the potential associated advantages of molecular-level control of their composition, biocompatibility, and simple bulk production and purification procedure that relies on phase separation and centrifugation.

## Procoagulant materials and therapies for clinical use

The development of biomolecular therapeutics along with hemostatic biomaterials such as sponges, sealants and foams for procoagulant use in humans has a long history. Early examples include injectable porcine skin gelatin tested clinically in the 1940s and the development of gelatin sponges in 1945 (Correll and Wise 1945). In terms of intravenous therapeutics for promoting coagulation and maintaining hemostasis, early examples include tranexamic acid developed in the 1960s and used until today (Tengborn et al. 2015). **Table ****1** tabulates procoagulant hemostatic agents for human clinical use, their mechanism of action, and commonly used trade names. This table is broadly split into local/topical products and intravenous products. The local/topical materials list includes several classes of macromolecules used for procoagulant effects including carbohydrates, structural proteins, peptides, inorganics, and synthetic polymers. The intravenous therapeutics section includes small molecule drugs along with recombinant and human-derived proteins and coagulation factors that have passed clinical trials for human use. This table can serve as a general reference list for the broad range of clinically available hemostatic agents in use today.

**Table 1:** Topical tissue sealants/adhesives and intravenous systemic agents for promoting coagulation and hemostasis

Procoagulants in clinical use	Mechanism of action and comments	Trade name (Supplier)	References
Local/topical			Reviews(Spotnitz and Burks 2008, 2010; Seyednejad et al. 2008; Panda et al. 2009; Spotnitz 2010, 2020; Chiara et al. 2018; Tompeck et al. 2020; Huang et al. 2020; Zhong et al. 2021)
Collagen	Microfibrillar collagen enhances platelet aggregation	EndoAvitine (BD), Costasis (Cohesion Tech.), Vitagel (Orthovita), AngioSeal (Terumo)	(Sirlak et al. 2003; Saif et al. 2011; Qerimi et al. 2013; Odermatt et al. 2017)
Cyanoacrylate	2 component chemical glues; indicated for incision closure, surface bleeds or mesh fixation	Dermabond (Ethicon), Indermil (US Surgical), Histoacryl blue (B. Braun)	(Vauthier et al. 2003; Belletrutti et al. 2008)
Fibrin glue (tip applicator or spray)	Generates artificial clot upon application; two component systems of fibrinogen + thrombin, optionally FXIII	Artiss (Baxter), Evarrest (Ethicon), Evicel (Ethicon), Tisseel (Baxter), TachoSil (Baxter), Raplixa(Mallinckrodt)	(Jackson 2001; Spotnitz 2014; Hanna et al. 2014; Bhagat and Becker 2017; Enns et al. 2020)
Gelatin	Chemically or enzymatically crosslinked	FloSeal (Baxter), GelFoam (Pfizer), SurgiFlo (Ethicon), LifeSeal (LifeBond), GRF (Microval)	(Saif et al. 2011; Chu et al. 2021)
Inorganics	Typically zeolites or nanoclays (e.g., smectite, kaolin); Absorb water and provide physical barrier	QuikClot (Teleflex)	(Alam et al. 2004; Pourshahrestani et al. 2016; Long et al. 2018; Yu et al. 2019)
Peptide-based	Electrostatic self-assembly of peptide fibers/hydrogels	PuraStat (Top Corp.)	(Giritharan et al. 2018; Subramaniam et al. 2019)
Poly(ethylene) glycol	Biocompatible branched or linear polymers; chemical crosslinking	Coseal (Cohesion Tech.), Duraseal (Integra), ProGel (BD), Spraygel (Covidien),	(Wallace et al. 2001; Torchiana 2003)
Polysaccharide-based	Typically chitosan, starch, cellulose, alginate, or hyaluronic acid	Endoclot (EndoClot +), HemCon (Tricol Biomedical), Celox (Celox Medical), Arista (BD), PosiSep (Hemostasis LLC), Oxycel (Betatech), Surgicel (Ethicon), Kaltostat (Convatec)	(Prei et al. 2016; Yang et al. 2017; Chen et al. 2020; Li et al. 2020; Nam and Mooney 2021)
Thrombin	Triggers clotting when applied locally; known immunogenic complications for bovine sources	Evithrom (human); Recothrom (recombinant); Thrombin JMI (bovine)	(Lundblad et al. 2004; Lawson 2006; Cheng et al. 2009)
Intravenous/systemic			Reviews(Lashof-Sullivan et al. 2013; Chan et al. 2015b; Hickman et al. 2018; Franchini et al. 2019; Butterfield et al. 2020; Ghosh et al. 2020)
Aminocaproic acid	Antifibrinolytic lysine analog; inhibits plasmin and plasminogen activator; less potent than tranexamic acid	Amicar (Amneal Pharma)	(Chauhan et al. 2004; Mahdy and Webster 2004)
Antihemophilic factor	Controls bleeding in congenital Factor VIII deficiency (hemophilia A); recombinant or human-derived	Advate (Baxter) Adynovate (Baxalta) Eloctate (Biogen Idec) Esperoct (Novo Nordisk) Helixate FS (Bayer) Hemofil-M (Baxter) Jivi *(PEGylated)* (Bayer) Koate DVI (Kedrion Biopharma) Obizur (Takeda)	(Schwartz et al. 1990; Lieuw 2017)
Anti-inhibitor coagulant complex	Non-activated mixtures of coagulations factors II, IX, X, and activated factor VII; used for bleeding control in hemophilia A or B	Autoplex T (Nabi Biopharmaceuticals), Feiba NF (Baxter) Feiba-VH (Takeda)	(Abildgaard et al. 1980; Leissinger et al. 2011; Rota et al. 2017; Carroll et al. 2020)
Aprotinin	Competitive inhibitor of plasmin; bovine-derived; safety concerns raised	Trasylol (Nordic)	(Landis et al. 2001; Mössinger et al. 2003; Steinmetzer et al. 2020)
Cryoprecipitate	Isolated from precipitated fraction of thawed plasma rich in Fibrinogen and factors VIII, XIII, and von Willebrand factor	Plasma Cryoprecipitate (Octapharma), Cryoprecipitate from national and regional blood banks	(Callum et al. 2009; Rourke et al. 2012; Rahe-Meyer et al. 2013; Levy et al. 2014)
Emicizumab	Bispecific antibody binds factors IX and X for bleeding control in hemophilia A patients; subcutaneous injection	Hemlibra (Roche/Genentech)	(Oldenburg et al. 2017; Blair 2019)
Fibrinogen	Human-derived pooled concentrates	Fibryga (Octapharma), RiaSTAP (CSL Behring)	(Ziegler et al. 2013; Lissitchkov et al. 2018, 2020)
FIX	Supports clotting for congenital factor IX deficiency (hemophilia B)	Octanine F (Octapharma), Mononine (CSL Behring), AlphaNine (Grifols), Bebulin (Baxter), Rixubis (Baxter), BeneFIX (Pfizer), Alprolix (Bioverativ), Ixinity (Medexus), Rebinyn (Novo Nordisk), Idelvion (CSL Behring)	(Powell et al. 2013; Nathwani et al. 2014)
FVIIa	Supports extrinsic coagulation in patients with hemophilia A or B	NovoSeven (Novo Nordisk), SevenFact (LFB)	(Abshire and Kenet 2004; Roberts et al. 2004; O’Connell et al. 2006)
FVIII	Recombinant or human donor-derived; indicated for hemophilia A	Nuwiq (recombinant), Octanate (Octapharma), Aafact (Sanguin)	(Schwartz et al. 1990; Mahlangu et al. 2014)
FX	Treats bleeding in FIX or FX deficiency	Coagadex (BPL), Factor X P Behring (CSL Behring)	(Shapiro 2017)
FXIII	Transglutaminase (recombinant or human-derived) for crosslinking fibrin clots in congenital FXIII-deficient patients	Tretten (Novo Nordisk), NovoThirteen (Novo Nordisk), Corifact (CSL Behring)	(Muszbek et al. 2011; Schroeder and Kohler 2013)
Human albumin	Maintains circulating blood volume	Albunorm (Octapharma) Albuminex (BPL) Kedbumin (Kendrion) Albuman (Sanguin)	(Fanali et al. 2012)
Prothrombin complex	Treats coagulation factor deficiency induced by vitamin K antagonist (e.g., warfarin) in adults with acute major bleeding; contains FII, IX and X	Kcentra (CSL Behring), Cofact (Sanguin)	(Leissinger et al. 2008; Sarode et al. 2013)
Tranexamic acid	Antifibrinolytic lysine analog; inhibits plasmin and plasminogen activator; oral or intravenous	Cyklokapron (Pfizer), Lysteda (Ferring)	(Goobie 2017; Wu et al. 2019; Goobie and Faraoni 2019)
von Willebrand factor	Treats bleeding in children and adults with von Willebrand disease	Wilate (Octapharm), Vonvendi (Baxalta)	(Berntorp et al. 2009; Franchini and Mannucci 2016)

## Biomolecular strategies for targeting fibrin

**Fig. 7:**
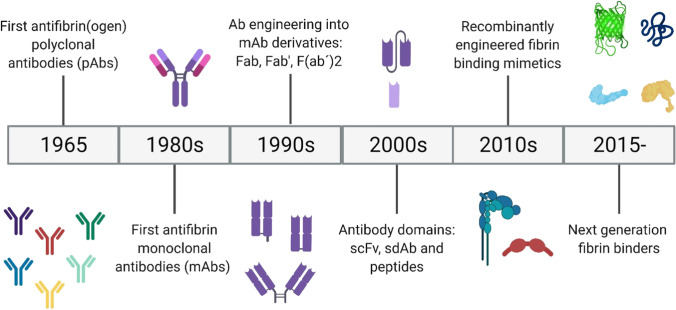
Timeline showing the development of fibrin binders. The timeline shows the development of antibodies and other binding scaffold formats used for the development of anti-fibrin molecules with the year or decade of first publication indicated. pAb, polyclonal antibody(Spar et al. 1965); mAb, monoclonal antibody; (Hui et al. 1983; Kudryk et al. 1984) Fab, monovalent antigen-binding fragment obtained after papain digestion of IgGs;(Raut and Gaffney 1996) Fab’, monovalent antigen-binding fragment obtained after pepsin digestion of IgGs and reduction in disulfide bridges (Flacke et al. 2001); F(ab)’2, divalent antigen binding fragment obtained after pepsin digestion of IgGs (Lugovskoy et al. 2004); scFv, single-chain variable fragment (Yan et al. 2004; Putelli et al. 2014); sdAb, single domain antibody/nanobody (Brown et al. 2014). Synthetic peptides with fibrin binding ability comprise linear short sequences (Starmans et al. 2013a; Wu et al. 2013) while cyclic peptides that may include D-amino acids or unnatural amino acids are circularized by Cys-Cys disulfide bonds (Kolodziej et al. 2012; Obermeyer et al. 2014). Antifibrin mimetics include molecules that naturally bind fibrin(ogen) or to their complexes that have been engineered for fibrin targeting (Ezov et al. 1997; Wu et al. 2003; Makogonenko et al. 2007; Klegerman et al. 2008; Martino et al. 2014; Litvinov et al. 2016; Briquez et al. 2017; Ghaheh et al. 2019). Next-generation fibrin binders include novel scaffolds that can be evolved for fibrin affinity and engineered for additional therapeutic features such as pro- or anticoagulant activity or image contrast (Liu et al. 2008; Fujita et al. 2018)

The first reports of fibrin binding antibodies for theranostic applications focused on localizing thromboses using cross-species polyclonal anti-fibrinogen antibodies isolated from rabbits immunized with dog fibrinogen (Spar et al. 1965). Rabbit antisera were used to label fibrin in clots; however, the antibodies did not discriminate between fibrin and fibrinogen. Two decades later, the development of hybridoma technology (Köhler and Milstein 1975) and a deeper understanding of the fibrin polymerization and fibrinolysis pathways helped in the development of the first monoclonal antibodies with high affinity and specificity to fibrin (Fig. 7). In the sections that follow, we provide an overview of the development of fibrin binders and the engineering strategies used for isolation.

### Monoclonal antibodies (mAbs)

Several early studies reported engineering mAbs specifically targeting fibrin. For example, Rylatt and colleagues developed the first mAb for the D-dimer antigen. Mice were immunized with the fibrin fragment D-dimer, or other preparations of cross-linked fibrin degradation products (Rylatt et al. 1983). Hui et al. synthesized a heptapeptide mimic of the amino terminus of the fibrin β chain to serve as a unique fibrin antigen based on the rationale that the amino terminus is exposed after thrombin cleavage and constitutes a conserved antigen unique to fibrin and not fibrinogen. Mouse immunization and hybridoma cell line development yielded the 59D8 mAb, which was found to bind human fibrin in the presence of fibrinogen, both in vitro and ex vivo (Hui et al. 1983). Following this strategy, Kudryk et al. developed the T2G1S mAb (Kudryk et al. 1984), which together with the 59D8 mAb constitutes a gold standard for fibrin targeting in current clinical applications and commercial antifibrin antibodies (Khaw 1999). Finally, Kanke and colleagues developed the 64C5 mAb to image pulmonary embolisms (Kanke et al. 1991). Altogether these works paved the way for the traditional generation of anti-fibrin monoclonal antibodies, usually in an IgG format.

Another significant aspect of anti-fibrin mAbs is their major influence in the design and engineering of diagnostic and therapeutic delivery technologies for thrombolytics, contrast/imaging agents or other active biomolecules. Runge et al. reported a fibrin-selective recombinant molecule that was generated by coupling a high-affinity anti-fibrin antibody (59D8) with a single-chain urokinase-type plasminogen activator scuPA, which is a fibrin-selective plasminogen activator that does not directly bind fibrin but binds and activates fibrin-bound plasminogen (r-scuPA-59D8). This engineered thrombolytic agent exhibited catalytic activity identical to that of WT-scuPA and fibrin binding activity comparable to that of the native 59D8 mAb. r-scuPA-59D8 was found to be sixfold more potent than WT-scuPA in lysing human clots in vitro and 20-fold more potent in a rabbit jugular vein model of thrombolysis (Runge et al. 1991). Bos et al. demonstrated a similar concept in recruiting plasminogen activators to fibrin clots using bispecific mAbs to enhance thrombolytic activity (Bos et al. 1990). These efforts were among the first to use recombinant engineered proteins including mAbs and other molecular entities for clot-targeted interventions.

Although full-length IgG antibodies can have a long half-life in blood and can be isolated with high affinity and specificity for a single antigen, full length IgGs also have intrinsic drawbacks. At ~ 150 kDa, full-length IgGs are large in size and can elicit immunogenic responses in humans, especially when they are derived from animal immunization procedures. Moreover, mAbs may be prone to physical and chemical instabilities that lead to aggregation, sensitivity to temperature and pH, and loss of activity or potency (Wang et al. 2007; Stefanelli and Barker 2015). To circumvent these limitations, mAbs have been dissected into their constituent domains, initially through papain/ pepsin proteolysis and later using genetic engineering to produce either monovalent (Fab, scFv, single variable VH and VL domains sdFv) or bivalent fragments (Fab′2, diabodies, minibodies, etc.…) (Holliger and Hudson 2005). Many of these alternative scaffolds to mAbs have also been developed for fibrin binding activity, as outlined in the next sections.

### Antibody derivative fragments (Fab, Fab’ and F(ab)2)

With a molecular weight of around ~ 50 KDa, the antigen binding fragment (Fab) is one of the most common antibody derivative fragments used in protein engineering. Fab fragments consist of the antigen-binding domains of standard mAbs, containing a variable region of the heavy chain (VH), a variable region of light chain (VL), a constant region of heavy chain 1 (CH1), and a constant region of the light chain (CL). Fab’ refers to a fragment containing a disulfide bridge, whereas Fab refers to a fragment lacking the disulfide (Joosten et al. 2003), while F(ab)’2 s are divalent antigen binding fragment obtained after pepsin digestion of IgGs (Lugovskoy et al. 2004). Typically, Fab fragments are produced via enzymatic/chemical cleavage of a full-length IgG using papain, pepsin and ficin followed by disulfide reduction (Crivianu-Gaita and Thompson 2016). Protease cleavage to generate Fab fragments requires large quantities of starting materials; therefore, single-chain Fab fragments (scFab) were developed using genetic engineering. Hust et al. showed that scFab fragments exhibit superior antigen-binding ability compared to Fab fragments and compensate for some of the disadvantages of the soluble Fab production in *E. coli* (Hust et al. 2007).

Development of Fab fragments with affinity for fibrin was carried out by Greco et al. (Greco et al. 1993). They produced a monoclonal antibody (TRFl) and engineered it as an F(ab’)2 against the human fragment D-dimer of cross-linked fibrin to detect human atherosclerotic plaques free of macroscopically detectable thrombi on their surface. They raised mAbs against fibrin epitopes using purified D-dimers from cross-linked fibrin as the antigen and proved that TRFl-F(ab’)2 s possessed a high specific affinity for atherosclerotic plaques and to a lesser extent arterial fragments containing fatty streaks. It was suggested that such F(ab’)2 s could be a useful compound for the scintigraphic detection of atherosclerotic disease. In a similar fashion, Kamat and colleagues developed an anti-fibrin Fab’ (Tc-99 m-antifibrin) for use in immunoscintigraphy of deep vein thrombosis (DVT) (Kamat et al. 1996). They showed that the Fab format retained high affinity for fibrin epitopes following technetium-labeling chemistry and formulation enhancement including additives for labeling, bulking, and stabilization.

In subsequent years, several examples of antifibrin Fab fragments were reported, for example, a bispecific antifibrin-antiplatelet urokinase conjugate BAAUC consisting of a monovalent Fab’ from the antifibrin monoclonal antibody 59D8 (Hui et al. 1983) and a monovalent Fab’ from the anti-glycoprotein GpIIb/IIIa monoclonal antibody 7E3. This compound was found to lyse both fibrin-rich and platelet-rich thrombi with high efficacy. The in vitro platelet aggregation and clot lysis activity of different urokinase constructs were also compared alone, fused to a full-length IgG, fused only to either one of the monospecific Fab, and finally as a bispecific construct. The bispecific construct displayed the highest activity, inhibited platelet aggregation and was proposed as an antithrombotic therapy (Ruef et al. 1999).

Flacke et al. developed a ligand-targeted paramagnetic MRI imaging agent with high avidity for fibrin using F(ab’)_2_ fragments derived from the previously reported anti-fibrin monoclonal antibodies (NIB 1H10, NIB 5F3) (Raut and Gaffney 1996) conjugated to acoustic contrast nanoparticles. By targeting two different epitopes on fibrin and formulating multivalent particles decorated with both Fabs, the effective affinity to fibrin was increased, allowing the sensitive detection of active vulnerable plaques (Flacke et al. 2001). Finally, Lugovskoy and colleagues used F(ab) fragments from parental antifibrin mAbs (II-3b and II-4d) to identify unknown epitopes in fibrin that could be targeted for inhibiting fibrin polymerization as antithrombotic therapies. They suggested for the first time that Fabs, as smaller entities than mAbs, could block more polymerization sites than full-length IgGs (Lugovskoy et al. 2004).

### Single-chain variable fragment antibodies (scFv)

Single-chain variable fragments (scFvs) consist of a fusion protein of the variable region of the heavy chain (VH) and variable region of the light chain (VL) domains of immunoglobulins, connected with a short linker peptide of ~ 10 to ~ 25 amino acids. The order of the domains can be either VH-linker-VL or VL-linker-VH. One advantageous feature is that scFv fragments can be expressed in both bacterial (i.e., *E.coli*) and mammalian systems (i.e., CHO or HEK293 cells) (Kramer et al. 2002).

There are a few examples of fibrin-specific scFvs that have shown promising results for targeting clots. Song et al. reformatted antifibrin mAbs from a hybridoma cell line 8E5 to produce an scFv and (scFv)_2_ recombinantly in *E. coli*. They achieved high fibrin specificity by using a synthetic heptapeptide GHRPLDKC, which is a defined epitope of the B chain of fibrin, as it is a fibrin-unique antigen exposed on the surface of fibrin but not on fibrinogen. Moreover, they proved that the scFv retained the fibrin binding activity of the parent mAbs while the (scFv)_2_ showed stronger binding than the single scFv (Song et al. 1997).

Karlheinz and colleagues followed a similar strategy by recycling and editing the 59D8 antifibrin mAb and genetically formatting it into an scFv. Its affinity was matured using phage display in 10 rounds of panning on the synthetic Bβ15-22 peptide of fibrin (β-peptide). To engineer an anticoagulant that selectively localizes and acts at the clot site, Karlheinz genetically fused the anticoagulant peptide hirudin to the C-terminus of the variable region of the scFv light chain. To release the biologically active N-terminus of hirudin at the site of clotting, a factor Xa protease recognition sequence site was introduced between scFv_59D8_ and hirudin. This recombinant anticoagulant inhibited clot growth in vitro more efficiently than native hirudin. In addition to binding fibrin, the fusion protein inhibited thrombin only in the presence of factor Xa by releasing the hirudin antithrombotic N-terminus upon factor Xa-mediate cleavage. This anticoagulant was preferentially active at bleeding sites and served as the basis for a novel pharmacological approach (Peter et al. 2000). Further work from this group focused on modifications of this system by coupling a Factor Xa inhibitor tick anticoagulant peptide (TAP) N-terminally to scFv_59D8_ (Hagemeyer et al. 2004).

Another approach using fibrin-targeted scFvs was taken by Yan and colleagues, whereby an scFv was developed to bind fibrin clots using the human single fold scFv libraries I + J (Tomlinson I + J). This effort is one of the first to use a human synthetic scFv-phage display library to select scFvs specifically against human fibrin clots. The purified scFv did not bind to fibrinogen in ELISA tests, and its fibrin binding ability was concentration dependent, suggesting a specific and unique conformational epitope in fibrin (Yan et al. 2004).

A recent development in antifibrin scFvs came from Putelli and colleagues (Putelli et al. 2014), who used antibody phage display to isolate binders to the N-terminal peptide of the fibrin α-chain. They panned a naive phage library and after selecting an enhanced binder with high affinity (K_D_ = 44 nM) to fibrin but not to fibrinogen or N-acetylated fibrin peptides, they further affinity matured the VH domain yielding an antibody termed AP2. AP2 was engineered and expressed as an scFv, a small immune protein and an IgG that were all found to inhibit fibrin clot formation in a concentration-dependent manner.

### Single domain variable fragment antibodies (sdFv)

Variable domain-like recognition motifs (sdFv) or single domain antibodies(Holliger and Hudson 2005) are naturally occurring small (~ 15 kDa) antibodies derived from the heavy chain-only antibodies found in camelids (VHH from camels and llamas), and cartilaginous fishes (VNAR from sharks). A commercial name was coined by the company Actelion, which popularized the term “nanobodies” for VHH and VNAR scaffolds. Nanobodies are useful alternatives to conventional antibodies due to their small size, high solubility and stability. Nanobodies can be recombinantly expressed in bacteria or yeast cells. In addition, phage-, ribosome-, and/or mRNA/cDNA display methods can be used for the efficient generation and maturation of these binders in vitro.

Employing phage biopanning against polymerized fibrin along with negative selection/competition with soluble fibrinogen, Barker and colleagues (Brown et al. 2014) isolated several fibrin-specific scFv and sdFv binders. The nanobody designated H6 bound fibrin with ~ 200 nM affinity with only weak binding to fibrinogen. This work exploited the avidity of gel particles decorated with H6 sdFvs to reduce bleeding times in rats on par with FVIIa, and better than infused fresh platelets.

### Synthetic peptides

Peptides, typically between 10 and 30 residues long, are the shortest sequences that exhibit binding activity and possess intrinsic advantages over antibody fragments in certain applications. The small size confers rapid clearance from the bloodstream which can be advantageous in imaging applications. Peptides also have low production costs and are frequently non-immunogenic. However, the length constraints can limit binding affinity to the micromolar range in many cases. Among the first to develop peptides to bind fibrin or fibrinogen, Kawasaki et al. (Kawasaki et al. 1992) reported that N-terminal tetrapetide analogs of the fibrin α-chain GPRP can inhibit fibrin polymerization and thrombin activity. Several analogs were synthesized and described as potent anticoagulants. Based on this sequence, Soon and colleagues (Soon et al. 2010) created engineered proteins that use the interactions between N-terminal fibrin knobs that bind C-terminal pockets of the fibrin network for protein delivery within fibrin matrices using the GPR motif and derivatives. They confirmed that GPRP sequence confers specific binding ability of fusion domains to fibrin(ogen).

In related work, the Ruoslathi group (Pilch et al. 2006; Simberg et al. 2007) used ex vivo and in vivo phage selection to discover several tumor-related fibrin-binding peptides. The CREKA peptide was found to home to tumor sites in vivo in transgenic breast cancer mouse models. Analysis suggested the peptide bound to a neoepitope formed due to the interaction between fibrin and fibronectin (FN), as it colocalizes with anti-fibrinogen antibodies in atherosclerotic plaques, but it does not bind tumors in knocked down fibrinogen or knocked down FN mice. Ruoslathis’s peptide has undergone preclinical and early clinical trials for the detection and targeted delivery of antithrombotic agents to clots formed in lung, prostate and glioma tumors, as well as to atherosclerotic plaques. Building on this, Peters and colleagues developed modular multifunctional micelles that contain a fibrin targeting CREKA peptide, a fluorophore, and, when desired, a drug component in the same particle to target atherosclerotic plaques (Peters et al. 2009; Agemy et al. 2010). Besides the CREKA peptide, the CLT-1 peptide with the sequence CGLIIQKNEC binds to fibrin–FN complexes as well. Although the epitope has not been confirmed, it is presumed that CTL-1 binds FN as suggested by minimal homing to tumors in fibrinogen knocked down mice, but not in FN-knocked down mice. In similar applications as the CREKA peptide, CTL-1 has been conjugated to radioisotopes for imaging tumors, atherosclerotic plaques, and fibrotic tissue in vivo (Pilch et al. 2006; Chow et al. 2013; Wu et al. 2013, 2014).

Cyclic peptides were developed in the early 2000’s by several researchers working for EPIX Medical Inc. One of the first synthetic peptides, EP-1873, is a fibrin-specific MRI contrast agent composed of a constrained six amino acid cyclic peptide core and four Gd-DTPA (diethylenetriami-nepentaacetic acid) chelates. The agent binds intact fibrin selectively without binding to circulating fibrinogen, allowing the detection of acute thromboembolic events throughout the body. Their clinical suggestions for potential thrombus detection in acute coronary syndromes and stroke led to preclinical trials of this compound (Wiethoff et al. 2003; Botnar et al. 2004). Recent work related to the evolution and development of novel agents based on EP-1873 peptides led to the discovery of EP-2104R, which is amenable to both MRI and NMR-based detection techniques (Sirol et al. 2005; Overoye-Chan et al. 2008). While this work focused on EP-2104R effectiveness for imaging, initial studies of the binding affinity of the peptide to fibrin were carried out on human plasma. Subsequent versions of EP-2104R have been developed from the Caravan lab ((Sirol et al. 2005; Overoye-Chan et al. 2008) (Nair et al. 2008); (Kolodziej et al. 2012)(Sirol et al. 2005; Overoye-Chan et al. 2008). The EP-782a and EP-821 peptides, both derivatives of the EP-2104R peptide, bind with low micromolar affinity to two sites on fibrin. Nair et al. (Nair et al. 2008) demonstrated that their monovalent constructs created by chemoselective ligation bind to two sites in fibrin and that their fibrin affinity is higher than the monovalent constructs. In related work, Ciesienski and colleagues developed three fibrin-targeted FBP1, FBP2, FBP3 peptides and used them as positron emission tomography (PET) probes for thrombus imaging (Ciesienski et al. 2013). Finally, Marinelli et al. developed and patented FibPep (Marinelli et al. 2015), a SPECT imaging probe for fibrin with an 11 In-labeled fibrin-binding peptide (Starmans et al. 2013a, 2013b).

In other work based on peptide phage display technology, Kolodziej et al. isolated cyclic peptides with low micromolar affinity for fibrin. Panning counterselection campaigns by incubating phages with fibrinogen eliminated non-specific binders while positive selection panning rounds against fibrin and immobilized DD(E) fragments were used to isolate small cyclic peptide families, referred to as Tn6, Tn7, and Tn10, that differ in the size of the central disulfide-linked macrocyclic ring and the sequences (Kolodziej et al. 2012). In all three of these peptides classes (Tn6, Tn7, and Tn10), the disulfide bridge is critical for fibrin binding (Kolodziej et al. 2012; Oliveira and Caravan 2017). Tn6 and Tn7 peptides were used as conjugates together with MRI contrast agents (Stefanelli and Barker 2015). Later, it was discovered that EP-2104R belongs to the Tn6 family (Oliveira and Caravan 2017). The Caravan group further developed an elegant strategy for fibrin targeting using a modified EP-2104R. Activated platelets overexpress the enzyme disulfide isomerase (PDI). Since PDI catalyzes disulfide exchange in the integrin α_2B_β_3_ that allows the platelet to bind fibrinogen, they hypothesized that in the presence of activated platelets and PDI, the peptide would undergo disulfide formation and cyclization, producing a fibrin targeting probe in situ. A linear EP-2104R analogue prodrug had no affinity for the fibrin DD(E) fragment, but in the presence of PDI, disulfide rearrangement re-established fibrin binding properties (Oliveira and Caravan 2017).

Another work on peptides by Stabenfeldt et al. employed molecular dynamic simulations to provide insight into the knob structural features that govern fibrin knob/hole binding interactions (Stabenfeldt et al. 2010). The group proposed knob design criteria for developing more potent binding peptides that significantly disrupt native knob/hole interaction. They proved the principle by developing a novel knob peptide mimic, GPRPFPAC that shows the highest reported affinity to the polymerization holes domains due to a unique element (Pro-Phe-Pro) that enhanced the association rate.

Avidity engineering has also been used to enhance binding of small peptides to fibrin. Obermeyer et al. (Obermeyer et al. 2014) proved this concept by conjugating 90 copies of a fibrin targeting GPR tripeptide to the exterior protein shell of bacteriophage MS2 capsids for fibrin targeting and imaging. The ability of peptide multivalency to enhance fibrin binding was shown through inhibition of clot formation at effective concentrations over ten-fold lower than the monomeric peptide alone.

### Engineered antifibrin mimetics

Several naturally occurring peptides, proteins, and enzymes have fibrin-binding activity, and these modules can be used for rational design of molecular systems targeting fibrin. Particularly plasminogen (Wiman and Collen 1978), plasminogen activators, and plasmin inhibitors can be emulated or exploited to create artificial fibrin-binding proteins.

Plasminogen heavy chain A contains 5 kringle domains while the light chain B is the serine protease domain (Fig. 2c) (O’Reilly et al. 1994). The 5 kringle domains display various levels of affinity for cross-linked fibrin, with kringle-1 having the strongest affinity followed by kringle-4 (Lerch et al. 1980). Menhart et al. recombinantly produced these domains in E. coli to test fibrin binding activity (Menhart et al. 1991). Wu and colleagues then developed a thrombolytic agent by fusing a Kringle-1 domain from human plasminogen to the C-terminal end of staphylokinase in a construct called SAKM3-L-K1 (Wu et al. 2003).

A frequently mimicked natural molecule is tissue-type plasminogen activator (tPA). Runge and colleagues (Runge et al. 1991) combined a high-affinity anti-fibrin mAb, 59D8, with a low molecular mass (32 kDa) single-chain urokinase-type plasminogen activator scuPA. The 103-kDa r-scuPA-59D8 protein was 6x more potent than WT scuPA in lysing a human plasma clots in vitro and 20 × more potent in a rabbit jugular vein model of thrombolysis. In related work, Klegerman et al. irreversibly inhibited human recombinant tPA (Activase®) with D-phe-L-pro-L-arg-chloromethyl ketone (PPACK) and conjugated it to intrinsically echogenic liposomes (ELIP) (Klegerman et al. 2008). More recent applications of tPA for fibrin targeting are from Taheri et al. (Taheri et al. 2016), who imparted fibrin-binding ability to the fibrinolytic agent streptokinase by fusing the tPA Kringle 2 domain to the streptokinase N-terminal domain and showing that the chimeric streptokinase exhibited stronger fibrin-specific activity compared to wild-type.

Another important natural protein that binds fibrin and modulates fibrinolysis is the inhibitor α2-antiplasmin (α2AP). α2AP is a primary inhibitor of plasmin-mediated fibrinolysis that is covalently cross-linked by FXIIIa to polymerized α-chains of fibrin and competitively abolishes plasmin digestion (Sakata and Aoki 1980). The fibrin recognition sequence in α2AP was characterized as the N-terminal sequence N_13_QEQVSPLTLLK_24_. This short peptide retains fibrin affinity and thus, has been used for the detection of nascent clots (Tung et al. 2003), for wound repair (Geer et al. 2005; Liang and Andreadis 2011; Sacchi et al. 2014), and for thrombolytic activity (Robinson et al. 2000; Stefanelli and Barker 2015). Briquez and colleagues reported the use of α2AP peptide incorporated into a human-derived plasmin inhibitor. Based on sequence homology to aprotinin, they identified the Kunitz-type protease inhibitor (KPI) domain of human amyloid-β A4 precursor protein as a lead candidate. They engineered the α2AP peptide into KPI variants to colocalize to fibrin via covalent binding through Factor XIIIa transglutaminase-mediated ligation. The engineered KPI variants significantly slowed plasmin-mediated fibrinolysis in vitro as compared with aprotinin (Briquez et al. 2017).

FN is an adhesive extracellular matrix protein that interacts with surface receptors on fibroblasts, neurons, phagocytes, and bacteria during wound healing and also has binding affinity for fibrin. The FN multidomain polyprotein consists of two disulfide-linked chains. Each chain contains homologous type I (finger), type II, or type III domains that are grouped into functional regions including fibrin-binding (Fib-1 and Fib-2), collagen-binding, cell-binding, and heparin-binding regions (Makogonenko et al. 2007). The N-terminal domain of FN has a fibrin-binding site and a site for covalent FXIIIa-mediated cross-linking to fibrin, which served as a basis for an engineered thrombus imaging agent described by Rosentall and Leclerc, who used the Fib-1 fibrin binding domain labeled with In-111 to detect fresh clots in patients (Rosenthall and Leclerc 1995). Ezov and colleagues used the fibrin binding properties of the N-terminal fibronectin domain by radioactively labelling the 5-finger FN domains and using them for the diagnosis of venous thrombosis (Ezov et al. 1997). Finally, Makogonenko et al. produced Fib-2 regions and tested interactions with recombinant αC-domains of fibrin(ogen) (Makogonenko et al. 2007; Stefanelli and Barker 2015).

As critical molecules for tissue repair with many interactions with the ECM including fibrin(ogen), growth factors (GFs) have also been used for fibrin targeting. Martino and colleagues found that a domain from placenta growth factor-2 (PlGF-2_123–144_) binds with high affinity yet promiscuously to the ECM proteins FN, vibronectin, tenascin C, osteopontin, collagen and fibrin(ogen). They fused PlGF-2_123–144_ to several GFs (vascular endothelial growth factor–A, platelet-derived growth factor–BB, and bone morphogenetic protein–2) to generate GFs variants with affinity for ECM proteins. These PlGF-2_123–144_-GFs aided the repair of chronic wounds and bone defects, surpassing the effects of the wild-type GFs along in rodent models. The group suggested that coupling an ECM binding mimetic domain may be useful for targeting fibrin in several regenerative medicine applications (Martino et al. 2014).

### Miscellaneous fibrin binders

In this section, we cover miscellaneous macromolecules with affinity for fibrin. For example, Fujita et al. showed that a thrombin-binding DNA aptamer called 29TBA with the sequence 5’ -AGTCCGTGGTAGGGCAGGTTGGGGTGACT- 3’ was entrapped in fibrin gels during the hydrogel polymerization catalyzed by thrombin. Fujita showed that 29TBA tightly bound thrombin (K_D_ ~ 0.29 nM) and suppressed thrombin activity down to 65%; however, this activity level was still sufficient for catalyzing fibrin assembly and thrombin entrapment, providing a possible approach for entrapment of growth factors, antibiotics, or anti-tumor drugs and their controllable release from fibrin gels (Fujita et al. 2018). Liu et al. reported a method for purifying fibrinogen based on the affinity of *Staphylococcus aureus* clumping factor A to fibrinogen. Clumping factor A (ClfA) is a cell wall-anchored protein with af­finity­ to the C-terminus of the γ-chain of fibrinogen. Active ClfA was genetically fused with the C-terminus of glutathione–S-transferase (GST) and used as an affinity­ ligand to isolate fibrinogen using affinity separation. ClfA_221–550_ is cross-reactive with both rat and mouse fibrinogen (Liu et al. 2008). Although the approach is similar to fibrin binding mimics, the idea of using adhesin domains from pathogenic bacteria with affinity for fibrin(ogen) opens the door for novel scaffold engineering for fibrin binding. There are many *S. aureus* and other bacterial adhesive proteins with affinity for fibrin; therefore, many scaffolds are potentially available.(Foster et al. 2014; Liesenborghs et al. 2018).

All the above-reported binders that target fibrin have been employed mainly as diagnostic tools where fibrin polymerization plays a major role in the pathology. For example, in cardiovascular diseases, fibrin accumulates in atherosclerotic plaques and serves as a marker for localizing early thrombi before myocardial infarction occurs. Imaging techniques that target polymerized fibrin, such as contrast-MRI, Doppler ultrasound, and angiography, also benefit from high-affinity fibrin binding proteins in diagnosis of clotting disorders. Here, either hemostats or thrombolytics that minimize potentially severe hemorrhage or thrombosis with high specificity for fibrin and not fibrinogen are needed. The same high specificity is used to target fibrin formation caused by inflammatory pathologies, such as rheumatoid arthritis, autoimmune disorders, or sepsis. More serious conditions can also be diagnosed, as fibrin can be a biomarker for central nervous system disorders like multiple sclerosis or as an oncological marker for certain types of cancer, where fibrin deposition is related to tumor development and metastasis. The main obstacle within the development of fibrin binders is to completely abolish non-specific binding to fibrinogen while retaining a high affinity for fibrin. This challenge has been tackled by using specific processed peptides unique to fibrin as antigens. A better understanding of fibrin polymerization and kinetics led to high-affinity mAbs and sdAbs with negligible binding to the fibrinogen precursor.

Nowadays, fibrin binders have been exploited for several purposes: for diagnostics, small binders like scFvs, sdAbs, peptides, and alternative scaffolds have been employed mainly because of the short half-life in the bloodstream, and also due to the high volume of distribution that generates better contrast for imaging and detection. Conversely, if a longer and more potent therapeutic effect is needed, molecules with higher molecular weight like Fab fragments or mAbs are used to extend the half-life. Next-generation fibrin binders will likely arrive as modular systems relying on antibody reformatting technology and immunoengineering methods to construct more efficient fibrin-targeting strategies that can ultimately reach clinical stages.

## Conclusion

With this broad review, we have provided background knowledge and summaries of specific studies on the topics of fibrin and coagulation proteins from a biophysical perspective. We have summarized what is known about the molecular pathways governing primary and secondary hemostasis with an emphasis on molecular and structural mechanisms. We described a series of related single-molecule studies using force-based assays with optical tweezers and atomic force microscopy investigating force activated conformational changes, including mechanical unfolding and activation of binding. Next, we provided an extensive list of clinical biomaterials and engineered therapeutics that serve to support coagulation and clotting in human patients. We described the biophysical properties of fibrin hydrogels from a soft mechanics perspective and then outlined anti-fibrin antibodies, peptides, polymers and antibody mimetics. Based on the importance of coagulation and clotting in a variety of pathophysiological processes, future work is poised to improve our understanding of the basic molecular processes underlying coagulation as well as develop novel formulations of engineered molecular systems capable of interfacing with the coagulation system in a controlled way.

## Data Availability

n/a.

## Code availability

n/a.

## Declarations

### Additional declarations for articles in life science journals that report the results of studies involving humans and/or animals

n/a.
